# Mechanoluminescence, thermoluminescence, optically stimulated luminescence and photoluminescence in SrAl_2_O_4_:Eu micro- and nanophosphors: effect of particle size and annealing in different atmospheres†

**DOI:** 10.1039/d3ra02514d

**Published:** 2023-08-29

**Authors:** Lucky Sharma, P. D. Sahare

**Affiliations:** a Department of Physics & Astrophysics, University of Delhi Delhi 110 007 India pdsahare@physics.du.ac.in

## Abstract

SrAl_2_O_4_:Eu in microcrystalline form was prepared by a combustion method. The formation of the material in a single phase was confirmed by XRD analysis. The material was crushed and sieved to get particles with different particle size ranges. It was further ball milled for 1–7 days to get particles in the nanosize ranges. The broadening of the XRD peaks of the phosphor material in nanocrystalline form was used to determine average particle sizes. The shapes and sizes of these particles could also be seen in FESEM images. The materials thus obtained were annealed in reducing (10% H_2_ in Ar) and oxidizing (in air) atmospheres at different temperatures for 1.0 h. The increase in the mechanoluminescence (ML) intensity on annealing in a reducing atmosphere at different temperatures and decrease on annealing in an oxidizing atmosphere could be attributed to redox reactions. This was further confirmed by PL measurements. Mechanoluminescence (ML), thermoluminescence (TL), and optically stimulated luminescence (OSL) of the materials were studied. In all three cases (*i.e.*, ML, TL, and OSL), the intensities are found to decrease with the particle size. A large shift of approximately 20 °C in the main peak of TL glow curves of micro- and nanocrystalline materials shows a widening of the band gap due to the particle size effect. A decrease in piezoelectric constant (*d*_33_) and field (F V m^−1^) with particle size was also observed. The present systematic study of particle size effect (over a wide range of particle sizes) on ML has great importance from a technological and application point of view for developing stress sensors.

## Abstract 2

Mechanoluminescence set up for ML measurements using dynamic compressive load.

## Graphical abstract

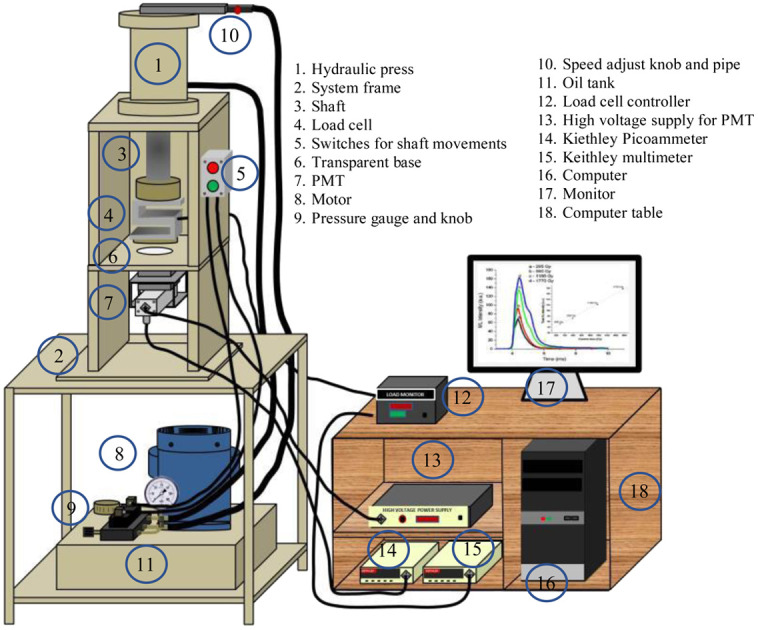

## 1 Introduction

Mechanoluminescence (ML) is the emission of light from organic or inorganic phosphor materials after applying mechanical stress, such as rubbing, grinding, scratching, cutting, bending, vibrating, stretching, and compressing.^1^ On the basis of the physical processes involved, it could be considered to be of two kinds, *i.e.*, deformation-ML (DML) and tribo-ML (TML). The former is produced during the deformation/fracture of solids, whereas the latter is due to contact phenomena occurring during the contact or separation of two dissimilar materials, such as tribo-electricity, tribo-chemical reactions, or tribo-thermal generation induced. DML could further be subdivided into three types, namely, fracto-ML (FML), plastico-ML (PML), and elastico-ML (EML). In FML, as the name suggests, luminescence is produced while creating new surfaces during the fracture of solids. In case of the PML, luminescence is produced due to plastic deformation of solids, while in the case of the EML, luminescence is generated by a process where neither of these two processes occurs, *i.e.*, neither the crystal is fractured nor there is any plastic deformation. It is generated due to elastic deformation. More details about these phenomena can be found elsewhere.^1–6^

Generally, all piezoelectric crystals with non-centro-symmetry exhibit ML; however, certain centrosymmetric, *i.e.*, nonpiezoelectric compounds also exhibit ML, but they have a nonpiezoelectric origin. There are a large number of inorganic and organic compounds that exhibit ML, including aliphatic and aromatic organic compounds, inorganic compounds, and minerals; besides these, there are several reviews on experimental and theoretical aspects including applications.^7–29^ A list of such known good mechanoluminescent phosphors could also be found in these review papers, including their method of synthesis. However, only a few of them, as mentioned in these reviews, such as, ZnS:Mn, SrAl_2_O_4_:Eu^2+^, SrAl_2_O_4_:Eu,Dy, SrAl_2_O_4_:Ce,Ho, Sr_3_Al_2_O_5_Cl_2_:Dy, CaZnOS:Mn^2+^, Ca_2_Al_2_SiO_8_:Eu^2+^, Ca_2_Al_2_SiO_7_:Ce SrCaMgSi_2_O_7_:Eu, ZnAl_2_O_4_:Mn^2+^, CaZr(PO_4_)_2_:Eu^2+^, ZnS:Mn,Te, Sr_3_Sn_2_O_7_:Sm, (Ba,Ca)TiO_3_, *etc.* are some of the highly intense ML phosphors and have found their applications for developing stress sensors.

Several review papers published in the literature show that not only bulk ML phosphors but also micro- and nanoparticles show very intense ML and even a single particle manipulation under an AFM allows one to produce light sources that could be useful for several photonic and other applications, including bio-applications.^30,31^ In some case(s), it is claimed that the ML intensity of nanoparticles is so intense that it could drive a solar cell and could also be used as an excitation source for specific applications.^32^ However, other reports show that the ML sensitivity is very low for smaller particle sizes owing to the large number of surface defect states. But, for many applications, such as bioimaging, counterfeiting, printing, and textiles, high ML sensitivity and particle size in the nano range are desired for better resolution and colour rendering. In addition, annealing at high temperatures in a reducing atmosphere is required to reduce Eu^3+^ to Eu^2+^ and also to prevent Eu^2+^ from oxidation for better sensitivity in europium-doped ML phosphor materials.^33^ It seems that the ML sensitivity depends upon several factors, including the method of synthesis, passivation of surface defects by capping, and redox reactions due to annealing and irradiation, and the best way could be to make a compromise between the particle size and sensitivity. The following discussion will clarify how such treatments could improve the sensitivity of the nanophosphors. Moreover, it is not only in ML but systematic studies in other luminescence phenomena, such as lyoluminescence (LL),^34^ thermoluminescence (TL),^35,36^ and optically stimulated luminescence (OSL)^37,38^ have revealed that on little compromise with the sensitivity, these (LL, TL and OSL) phosphors have great advantage for applications in radiation dosimetry for measurements of very high doses of high-energy radiations where the single crystals/microcrystalline phosphors saturate. This has been proved theoretically also.^39^ Similar things are possible for measuring very high values of stress using ML nanophosphors besides other important applications mentioned above.

As mentioned earlier, some of the synthesis methods of ML nanoparticles and different treatments improve their sensitivities, which are further discussed here. Xu *et al.* have developed ZnS:Mn film by physical vapour deposition on various substrates, such as metal plates (aluminum, and stainless steel), quartz, glass, and some ceramics (*i.e.*, Al_2_O_3_, SiC, Si_3_N_4_) and claimed that the high orientation and crystallization of the film give higher piezoelectric voltage on the opposite sides of the nano-sized grains of ZnS and provide higher ML intensity for dynamic imaging of stress distribution.^40^ The same group further developed a crystalline film consisting of nanoparticles onto various substrates and claimed that the ML is so intense to be seen by naked eyes.^41^ Tiwari *et al.* used mercaptopropionic acid (MPA) as a capping agent for surface passivation of ZnS:Cu nanophosphors. The surface defects/states are generally highly unsaturated due to the presence of strains, dangling bonds, *etc.*, and may act as luminescence quenching centers. Capping the surfaces of the nanophosphors removes such defects and does not affect the ML intensity of these materials drastically compared to their bulk forms. However, it may depend on the nature of the capping agent used.^42^ Rani and Sahare have studied the structural and morphological changes of the ZnS nanocrystalline ZnS system during the phase transformations of ZnS to ZnO by simple annealing in an air atmosphere at various temperatures and reported corresponding changes in band gap and PL spectra.^43^ Later, Wang *et al.* studied the ZnS:Mn microcrystalline system in a controlled O_2_ atmosphere and found the positive impact of S vacancies to improve the PL and ML characteristics. They further fabricated a device for dynamic stress imaging with excellent sensitivity using this material.^44^ From an application point of view, ML in nanostructured materials has already been used for various applications. For example, Sohn *et al.* have developed a mechanoluminescent ZnS:Cu/rhodamine/SiO_2_/PDMS hybrid sensor for applications like social infrastructure safety diagnosis, emergency guide lighting, and more importantly, for biomedical applications, such as diagnosis of motility and peristalsis disorders in the gastrointestinal tract.^45^ The ability to convert the input mechanical stimuli to either electric or light output is achieved by monolithically integrating a transparent single-electrode triboelectric nanogenerator (S-TENG) with a ZnS-based mechanoluminescence (ML) composite for its application in multifunctional wearable devices and artificial e-skins.^46–49^

Elastico-mechanoluminescent (EML) nanoparticles can be mixed with polymers to make a smart coating that allows viewing the stress distribution of an object. Elastico-luminescent nanoparticles will certainly enhance the space resolution and consequently provide more detailed information in the case of a tiny object. For example, Xu *et al.* have shown experimentally as well as by simulation that a controlled structure of α-SrAl_2_O_4_:Eu (monoclinic phase) nanoparticles giving strong EML could be used for high space resolution imaging for the visualization of stress distribution.^50^ In another study by Yamada *et al.*, there was an enhancement of adhesion and triboluminescent properties of SrAl_2_O_4_:Eu^2+^ films prepared by RF magnetron sputtering technique and giving post-annealing treatments.^51^ R. Sharma and U. Sharma attempted to prepare nanoparticles of SrAl_2_O_4_:Eu^2+^ nanophosphors using H_3_BO_3_ as flux and found that the concentration of the fluxing agent influences the luminescence properties of the ML nanophosphor.^52^ Li *et al.* have synthesized SrAl_2_O_4_:Eu nanoparticles in a desired phase by spray pyrolysis.^53^ Fujio *et al.* have shown that particle size and shape due to different synthesis methods could change the sensitivity and trap levels in their SrAl_2_O_4_:Eu phosphor material.^54^

Strontium aluminate (SrAl_2_O_4_) is found to be very unstable under the action of water. It tends to deteriorate when exposed to water to form hydroxides of the constituent elements. Therefore, if such rare earth-doped ML phosphors are used for maintaining the quality of big structures, their effectiveness would be compromised. Encapsulation methods, including coatings SiO_2_,^55,56^ Al_2_O_3_,^57^ TiO_2_,^58^ SrF_2_,^59^ and organic ligands,^60,61^ could be used for surface modification of phosphors to improve their luminescence properties.^62^ However, organic materials, such as polymers and plastics, are mostly permeable and fail to provide an impervious barrier against water. They should also be translucent, if not completely transparent. Also, the deterioration of the phosphor material would depend on the particle size as the surface area would increase with decreasing particle size. The interaction of such core–shell may also play an important role in the performance of the phosphor by increasing or decreasing the surface defects, and therefore, it is important to study the particle size effect on the ML of such phosphors.^63^ Terasaki *et al.* have come out with a novel idea of attaching TiO_2_ catalyst on SrAl_2_O_4_:Eu to produce a hybrid mechanoluminescent nanophosphor for its application as a catalyst even in the dark, *e.g.*, for removing toxins inside the body by applying physical stress.^64^ They also claim that a single ML particle of SrAl_2_O_4_:Eu could be used as an intensity-controllable light source in nW order.^65^

From the above discussion, it is evident that there are numerous applications of ML-based sensors and devices, and the nanophosphors have more advantages over their microcrystalline counterparts. There is hardly any field where such devices have not played their roles. Initially, it was thought that ML could be used in stress sensing, paints, emulsions, and devices to visualize active cracks on bridges and buildings for their maintenance,^66–68^ but now, there are ML fabrics and textiles that could show a colorful visualization just by wind flow, and there is no need of any power for such displays.^69,70^ There could be dynamic pressure mapping personalized handwriting for developing a high-level stealth security system.^71^ In the face of increasingly serious counterfeiting problems, there is a hard-pressed need to develop new kinds of advanced anti-counterfeiting mechanisms as fraudulent people already know about the existing systems, and new encryption patterns using ML could be developed to counter this problem.^72^ It has already been shown for a photocell system driven by ML for power generation.^73^ ML could also be used for printable skin-driven mechanoluminescence devices, data storage, and visual expression devices. In some of these systems, nanophosphors have also been used.^74,75^ The literature survey presented here shows that in spite of the need, the phosphor materials investigated to date are either in microcrystalline forms or a few have been studied in the nanocrystalline forms, and there is no systematic study on the particle size effect on the performance of ML phosphor materials. From an academic point of view, there is a need to study the surface states/defects and their passivation to improve the performance of the nanomaterials. This prompted and encouraged us further to take up this study.

In the present work, SrAl_2_O_4_:Eu ML phosphor material was prepared by a well-known combustion method using strontium and aluminum nitrates as starting materials and urea as fuel. It was crushed and sieved to get particles having different particle size ranges. It was further ball milled gently using a very low-speed ball milling for 1–7 days to get particles in the nanosize ranges. It was characterized by XRD and FESEM for confirmation of the crystalline structure, phase, and morphology for different particle sizes and their ML, TL, OSL and PL was studied. The material was also annealed in reducing (10% H_2_ in Ar) and oxidizing (in air) atmospheres at different temperatures for 1.0 h to improve the ML intensity by converting Eu^3+^ impurity ions into Eu^2+^ and also preventing the oxidation of Eu^2+^ into Eu^3+^. It was found that by annealing the material in a reducing atmosphere, the ML intensity increased substantially, and the reverse effect was observed by annealing in an oxidizing atmosphere. It is attributed to the redox reactions of the Eu-impurity as confirmed by the PL measurements. It was also observed that the annealing at higher temperatures agglomerated the nanoparticles into microcrystalline form and increased the ML intensity. It clearly shows that the intensity depends on the particle size. It was very interesting to see that the ML (linearly increasing compressive as well as impulsive load), TL, and OSL intensities decrease with the particle size decreasing. The decrease in piezoelectric constant (*d*_33_) and the piezoelectric field with the average particle size also show similar trends as the luminescence studies. These results show that studies on particle size effect are important from academic as well as application points of view and may further help in improving the performance of such phosphors by using different methods of synthesis, heat treatments, and techniques for passivation of surface defects.

## 2 Experimental

SrAl_2_O_4_:Eu (SAOE) phosphor was prepared by the combustion method using analytical grade strontium nitrate Sr(NO_3_)_2_, aluminum nitrate Al(NO_3_)_3_·9H_2_O as oxidizers, urea CO(NH_2_)_2_ as fuel, and europium nitrate Eu(NO_3_)_3_ as a source of the impurity. There are reports^66,67,76^ indicating that some other minority phases, like Sr_3_Al_2_O_6_ and Sr_4_Al_14_O_25_ having different Sr/Al ratios, could be formed due to lack of homogeneity in the system during the combustion process if the process is performed at lower temperatures; therefore, the combustion process was done at different temperatures, and it was optimized at 600 °C. It has also been reported in the literature that different oxidizers to fuel ratios (≥2) could form some undesired phases, such as SrCO_3_, due to the reaction of unreacted urea with Sr(NO_3_)_2_. Therefore, the fuel-to-oxidizer ratio was kept at 1 : 1.^77,78^ The stoichiometric composition of the materials in the chemical equation was calculated considering the total valency of the oxidizing and reducing elements, which serve as the numerical coefficients in the following chemical reaction:3Sr(NO_3_)_2_ + 6Al(NO_3_)_3_·9H_2_O + 20CO(NH_2_)_2_ → 3SrAl_2_O_4_ + 94H_2_O↑ + 20CO_2_↑ + 32N_2_↑

The weighed quantities of each nitrate and urea were crushed and mixed together in a mortar with little quantity of water for 1.0 h to form a thick paste. The resulting paste was transferred to an alumina crucible. The impurity (Eu(NO_3_)_3_) in an appropriate amount was also dissolved in a few drops of water and mixed thoroughly with the paste. The crucible was inserted in an open resistive furnace preheated at 600 °C. In a few moments, the combustion reaction starts, and the paste swells into a foam and ignites to burn itself. The materials having different impurity concentrations (0.05–2.0 mol%) were prepared to see the effect of impurity concentration. The formation of the material was confirmed by XRD using an X-ray diffractometer (Regaku, Japan, Model Ultima-4). The materials thus obtained were crushed gently and sieved to obtain particle sizes in different micrometer ranges (25–250 μm). The material having particle sizes below 25 μm was further processed through a ball milling machine (Metrex India, Model MLB-181) with polytetrafluoroethylene (Teflon) lined and horizontally fitted container using zircon balls (5.0 mm diameter) for 1–7 days. The material was annealed in oxidizing (air) as well as in reducing (10% H_2_ in argon) atmospheres at different high temperatures for 1.0 h to see the effect of annealing atmosphere and temperature. Small amounts of material were taken out after 1, 3, 5, and 7 days to obtain the particles in sub-nano to nano-ranges. Average particle sizes of the material were calculated using Scherrer's formula *D* = (*kλ*/*β* cos *θ*), where *D* is the size of the particle, *k* is known as the Scherer's constant (*k* taken here as 0.90), *λ* is the X-ray wavelength (1.5406 Å for Cu-K_α_ line), and *β* is the full width at half maximum (FWHM) of the diffraction peak (100). They were also calculated using other prominent XRD peaks, such as (2̄21), (220), and (031) and the average was taken. The morphology and particle sizes of the materials were also confirmed by FESEM. EDS measurements, elemental composition, and mapping were done using the EDS attachment (Ametek, USA, Model Octane Elect) to the FESEM equipment (Zeiss, Germany, Model Gemini-500).

Mechanoluminescence measurements by compression technique were taken using *‘homemade’* hydraulic machine fitted with a load cell, polymethyl methacrylate (Lucite) transparent base, and a broadband PMT (Hamamatsu, Japan, R-5108) to a light tight black-box. The applied load could be set and controlled using a load cell and controller (Adi Artech, India, Model 202101T and Nutronics, India, Model NI-2070B, respectively). The output of the load cell and the PMT current was recorded and stored in a computer in digital form using Keithley multimeter (Model 2100), pico-ammeter (Model 6485), IEEE-488 interface card, and Lab-VIEW software. The load was calibrated using different loads onto the load cell. A schematic sketch is as shown in Fig. S-1, ESI† file. An equal amount (approx. 50.0 mg) of the phosphor material was spread uniformly over the 2.0 cm^2^ circular area every time. The shaft was initially lowered down around 1.0 cm above the base at a higher speed and then continued with a uniform speed of ∼1.0 mm min^−1^. The ML intensity and load output were recorded simultaneously with time. Another ‘*home-made*’ set-up for impulsive ML measurements consists of a vertical steel pipe attached to a light-tight PMT housing and a Lucite transparent base as a sample holder over which loads of different masses could be dropped instantaneously using a thin weightless steel wire and a pulley. Another end of the vertical pipe was closed using a lid with a small hole for wire, making the whole system light tight. The PMT (Hamamatsu, Japan, R-5108) connected to a digital storage oscilloscope (Scientific, India, Model SMO1002ED) records the impulsive ML decay curves with time (in milliseconds) in digital form. Details could be found elsewhere.^1^ The samples were also irradiated to different doses of γ-rays from a ^60^Co radioactive source (dose rate 5.0 Gy min^−1^), UV radiation and thermoluminescence (TL) was taken using Harshaw TLD Reader (Model- 3500HT, Thermofisher Scientific Inc. USA). It was also found that the phosphor material gives TL without irradiation as well and therefore TL of materials without irradiation was also taken. For taking TL, approx. 5.0 mg of the irradiated phosphor sample was kept on the planchet (sample holder and heater) each time and heated with 5.0 K s^−1^. OSL measurements were taken on a TL/OSL Reader (Nucleonix India, Model TL/OSL-1008) fitted with PDM9107-CP-USB plug and play photon counting module. PL measurements were taken on a fluorescence spectrometer (Horiba Inc. Model Fluorolog 3-21). The dielectric constant and *d*_33_ piezoelectric constant measurements were taken on a broadband dielectric/impedance spectrometer (Novocontrol, Germany, Model α(alpha)-s) and *d*_33_ Piezometer (Pizotest Ltd. UK, Model Piezotest PM 300), respectively. The photograph of the glowing SAOE phosphor material during the application of the load was taken using Canon EOS 1500D 24.1 digital SLR camera with EF S18-55 II lens.

## 3 Results and discussion

### 3.1 Structural and morphological characterization

**Fig. 1:**
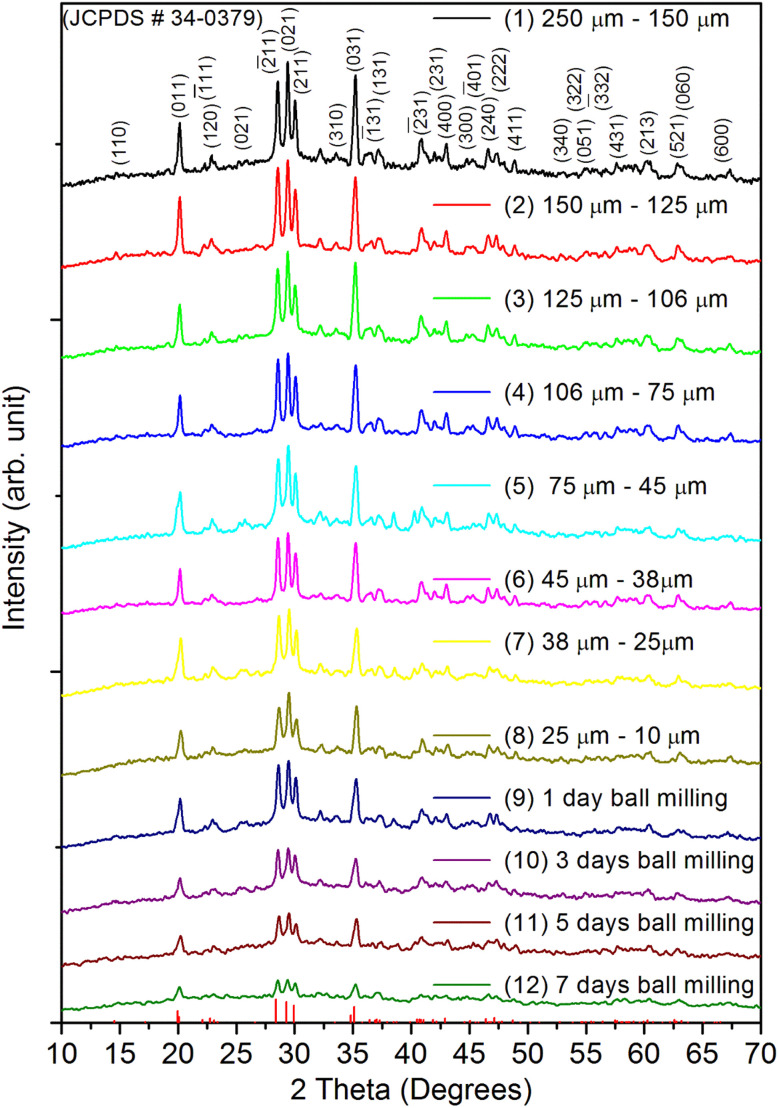
XRD patterns of all the materials in different micro- and nanocrystalline ranges: (1) 250–150 μm, (2) 150–125 μm, (3) 125–106 μm, (4) 106–75 μm, (5) 75–45 μm, (6) 45–38 μm, (7) 38–25 μm, (8) 25–10 μm, (9) sample after 1-day ball milling, (10) sample after 3-days ball milling, (11) sample after 5-days ball milling, (12) sample after 7-days ball milling. The data (XRD stick-pattern) using the data from the JCPDS file # 34-0379 is also given here for ready reference.

**Fig. 2:**
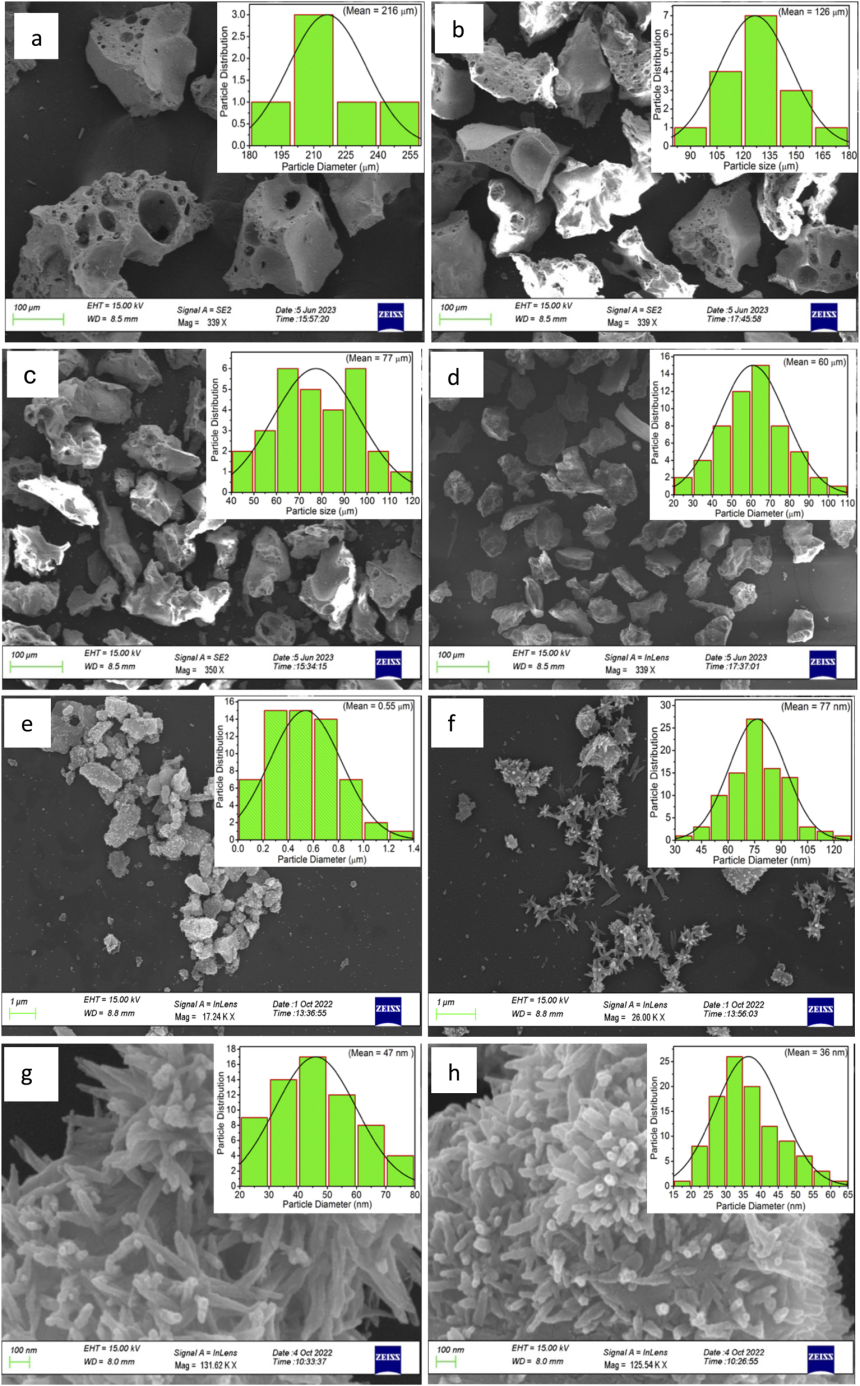
FESEM of the selected micro- and nanocrystalline materials: (a) 250–150 μm, (b) 125–106 μm, (c) 106–75 μm, (d) 75–45 μm, (e) 0.55 μm, (f) 77 nm, (g) 47 nm and (h) 32 nm. The particle distribution histograms are given for ready reference.

Fig. 1 shows the XRD pattern for the whole range of the particle sizes, *i.e.*, microcrystalline as well as nanocrystalline (∼250 μm to 30 nm) SrAl_2_O_4_:Eu (1.5 mol%) ML phosphor materials. The experimental data were theoretically fitted using Rietveld refinement (Fig. S-2, ESI file) and were also compared with the standard data available in the literature (JCPDF file # 34-0379).^79^ It could be seen in these figures that there is neither any change in the XRD patterns except the broadening of the peaks of nanocrystalline materials nor any change in the crystallinity of the material. For getting the material in different particle size ranges, the pristine material was crushed initially using an Agate mortar and pestle and sieved using standard test sieves of different sizes in the particle size ranges. The particle size distribution histogram and the morphology of these particles are shown in Fig. 2(a–h) insets. The powder having an average particle size below 25 μm was further processed through a ball milling machine for different time periods from 1-7 days to get the material in different nanocrystalline ranges. The average particle size of the nanophosphor was determined from the broadening of the most prominent and isolated peaks (2̄21), (220), and (031). The average particle sizes of the samples by ball milling for 1, 3, 5, and 7 days were found to be 32.0, 30.0, 28.0, and 25.0 nm by XRD broadenings and 550, 77.0, 47.0, and 32.0 nm as seen in FESEM images. The particles seen in the FESEM images (Fig. 2(e–h)) are of irregular shapes, and there could be grain boundaries amongst many particles, and hence, at submicron and nanosize levels, the values of particle sizes determined by two techniques, *i.e.*, FESEM and XRD, do not match. It could be seen in these images that it is not the particle size but the morphology of the particles also change. Initially, the bigger particles are of irregular shapes and porous in microcrystalline ranges but after they were ball milled gently in nanocrystalline ranges, they get transformed into more regular rod-shaped particles. However, the ball milling has also introduced strain/stress in the particles. The values of strain for different particle sizes have been determined from the Williamson–Hall plots (*β* cos *θ vs.* 4 sin *θ*) shown in Fig. S-13.† The slopes of the fitted lines give the values of strains. The determined values of strains were found to be 0.036, 0.361, 0.307, and 0.160 for the particle sizes 550 nm, 77.0 nm, 47.0 nm, and 32.0 nm, respectively. They were not determined for the microcrystalline materials as the XRD peaks are quite sharp. The porosity and the shapes and sizes of the phosphor materials might be playing an important role in their ML performances. The porosity in the microcrystalline material might help to deform the particles easily and polarize the electric charges for producing piezoelectric field and ML. As the particle size decreases, the surface-to-volume ratio increases, but the total area available to develop a sufficiently large piezoelectric field decreases for ML, resulting in low ML intensity.

### 3.2 Energy-dispersive X-ray spectroscopy (EDS)

The compositional study of SrAl_2_O_4_:Eu (1.5 mol%) for micro- and nanophosphors for the pristine sample was carried out by using EDS techniques. FESEM and atomic mapping are shown in Fig. S-3(A), S-3(B–F),† respectively, for particles in the particle size range of 250–150 μm. The EDS spectrum is shown in Fig S-3(G)† and a table consisting of the composition of the material in atomic% and weight% is also shown in its inset. Similar data for all the other particle sizes are also shown in Fig. S-4(A), S-4(B-F), and S-4(G), Fig. S-5(A), S-5(B-F) and S-5(G), Fig. S-6(A), S-6(B-F) and S-6(G), Fig. S-7(A), S-7(B-F) and S-7(G), Fig. S-8(A), S-8(B-F) and S-8(G), Fig. S-9(A), S-9(B-F) and S-9(G) and Fig. S-10(A), S-10(B-F) and S-10(G)† for the average particle sizes/ranges, 150–106, 106–75, 75–45, 0.55 μm, 77 nm, 47 nm, and 32 nm, respectively. The EDS provides clear evidence for the presence of strontium (Sr), aluminum (Al), oxygen (O), and europium (Eu) atoms in synthesized samples. The presence of peaks corresponding to Eu (one at around 1.1 keV and another at around 5.8 keV) in the EDS spectra confirms the presence of Eu in the SrAl_2_O_4_:Eu matrix of all the materials. A good homogeneity of the elements could also be seen in these figures.

### 3.3 Optimization of impurity concentration by ML studies by compressive technique

The mechanoluminescence (ML) of the samples with different impurity concentrations was studied by compressive technique and varying the load in the range of (0.0–1.0 × 10^3^ N). The ML glow curves are shown in Fig. S-11 (ESI file†). The variation of the ML peak intensity with the impurity concentration is also shown in the inset. It could be seen from the figure that the maximum ML intensity was found for the molar concentration of 1.5 mol% and decreases by increasing the impurity concentration further. The sample with this concentration, *i.e.*, SrAl_2_O_4_:Eu (1.5 mol%) was therefore used for further studies.

### 3.4 Effect of annealing temperature on the ML (compressive) peak intensity

SrAl_2_O_4_:Eu (1.5 mol%) was annealed in air at various elevated temperatures for 1.0 h. The results are shown in Fig. S-12 (ESI file†). It can be seen in the figure that the phosphor material shows maximum ML intensity after annealing at around 200 °C and then decreases by increasing the annealing temperature to higher values. This may be occurring due to two reasons; firstly, at lower temperatures up to 200 °C, the impurity might be getting dispersed uniformly in the matrix generating a greater number of traps. Secondly, there is a process of self-generation of traps on annealing that is not yet understood properly. However, at higher temperatures, the traps might be getting sufficient energy to get released and getting recombined with the luminescent centers during annealing. But, according to Kher *et al.*,^80^ ML is not only related to the traps but also to the movements of the dislocations and the luminescence is produced due to the interactions with defect centers and traps. This is also in line with the phase transitions under load from monoclinic (α-phase) to hexagonal (β-phase)^81^ where there would be movements of dislocations on phase transition. The impurity could enter in both Eu^2+^ and Eu^3+^ ionic states at virtually two different substitutional positions of Sr(1) and Sr(2), having effective coordination numbers 5.80 and 6.14. The charge compensation may be provided by the AlO_4_-tetrahedra.^82^ The annealing at higher temperatures in oxidizing and reducing atmospheres could induce redox reactions Eu^2+^ ↔ Eu^3+^, changing their ratios (discussed in more detail in Section 3.6). This would certainly affect the ML, TL, OSL, and PL intensity as well as the spectra. The atmospheric oxygen during annealing in air at high temperatures may get diffused in the matrix altering the composition or forming small clusters of new phase(s).^83,84^ The phase transition from monoclinic α-phase to hexagonal β-phase on heating in the range of 650–700 °C and also under a load to twinning pseudo-elastic deformation may enhance the accumulation of surface charges. On the basis of these results, Matsuo *et al.*^81^ have suggested the ML mechanism in SAOE that twinning pseudo-elastic deformation under a load creates a local electric field around the twin interface. The electric field releases the trapped electrons. Also, during deformation, the movement of dislocations excite the hole traps to release holes into the valence band. Holes then excite Eu^1+^ to produce Eu^2+*^, which then returns to the ground state by emitting a green light at around 520 nm.^83,84^ The traps may be regenerated by irradiation with UV-radiation.^85,86^ However, it does not explain the self-reproducible ML after annealing the material between 80 and 300 °C.^87–89^ There are, however, conflicting results found in the literature, Pateria *et al.*^88^ reported that all the traps are recombined during annealing in the dark at 350 °C for 30 min in their ZnS_1−*x*_(MnTe)_*x*_ material, and no ML was observed. On the contrary, Chandra *et al.* have suggested that the self-reproducible mechanoluminescence in ZnS:Cu and ZnS:Mn^2+^ arises as a contribution of drift current on the trap-recharging and could be attributed to the higher conductivity of semiconductors as compared to dielectric materials.^89^ According to Zhang *et al.*,^86^ though this explanation is plausible, it could be noted that many semiconductors do not show self-reproducible ML; for example, ML in ZnGa_2_O_4_:Mn^2+^, MgGa_2_O_4_:Mn^2+^ and ZnAl_2_O_4_:Mn^2+^.^90,91^ Rather, as mentioned earlier, it is more confusing that SrAl_2_O_4_:Eu^2+^ exhibits self-reproducible ML after annealing between 80 and 300 °C though it is a dielectric material. Therefore, more research is needed to understand the mechanism of self-reproducible ML. In the present study, we have tried to address this problem.

### 3.5 Particle size effect on the compressive ML intensity

**Fig. 3:**
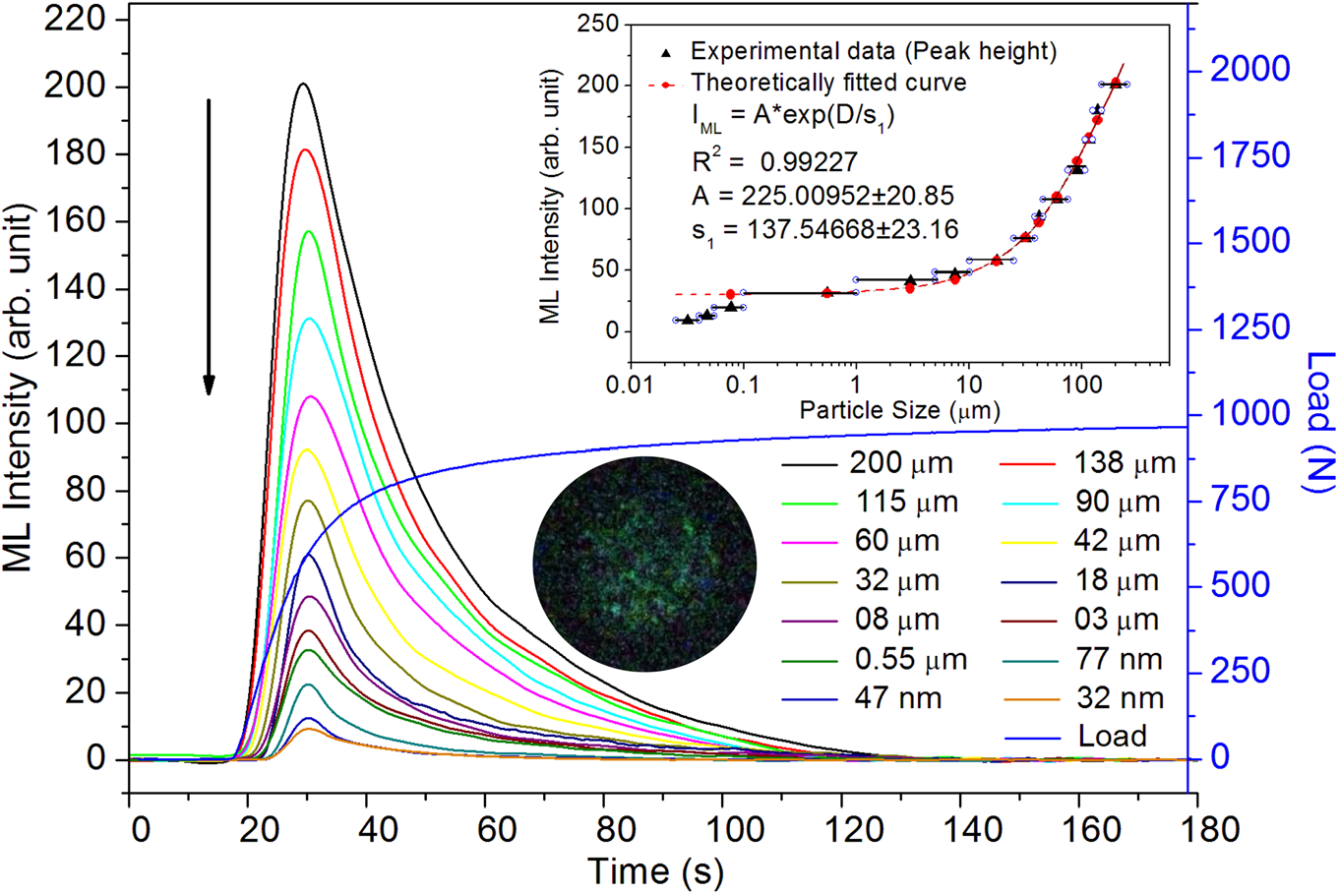
Variation of the ML (compressive) intensity with time for different particle size ranges. The variation of the intensity with the average particle size is also shown in the inset. The horizontal bars show the particle size range. The curve (intensity *vs.* particle size) has also been theoretically fitted and shows an exponential decrease in intensity with particle size decreasing. The equation with different parameters is also given in the inset. There is a very good fit up to the submicron size range. However, at nanoparticle size range as little deviation could be seen. The photograph of the captured ML glow is also shown in the inset.

As mentioned in the Experimental section, the material was crushed and sieved using mortar and pestle to get particles in different microcrystalline particle size ranges (250–25 μm). The powder below 25 μm was further ball milled to get particles in the nanoranges. The compressive and impulsive ML was taken on the ‘*home-made*’ systems as described earlier in the ‘Experimental’ section. The results are shown in Fig. 3. The variation of intensities with particle size is also shown in the inset of this figure. It was further theoretically fitted to find the relation of the intensity with the particle size. It could be clearly seen here that the plot of intensity with particle size shows an exponential decrease in the ML intensity with decreasing particle size. A detailed analysis of such behavior analogous to existing models^10,32^ is also given below.

**Fig. 4:**
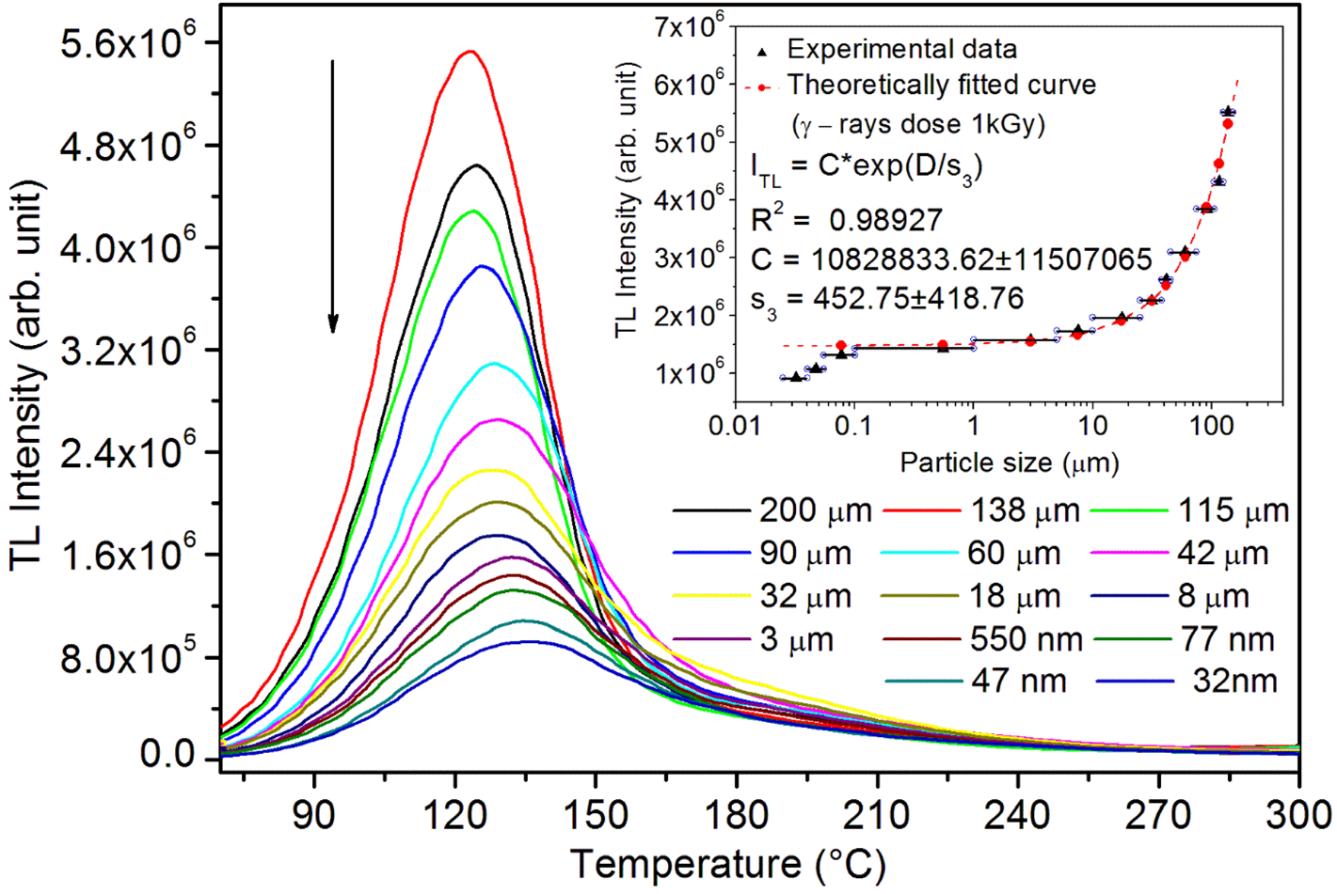
Variation of the TL intensity with temperature (TL glow curves) for different particle size ranges. The material was irradiated to 1.0 kGy dose of γ-rays from ^60^Co radioactive source. The variation of the intensity with the average particle size is also shown in the inset. The horizontal bars show the particle size range. The curve (intensity *vs.* particle size) has also been theoretically fitted and shows an exponential decrease in intensity with decreasing particle size. The equation with different parameters is also given in the inset. There is a very good fit up to submicron size range. However, at nanoparticle size range a slight deviation could be seen.

**Fig. 5:**
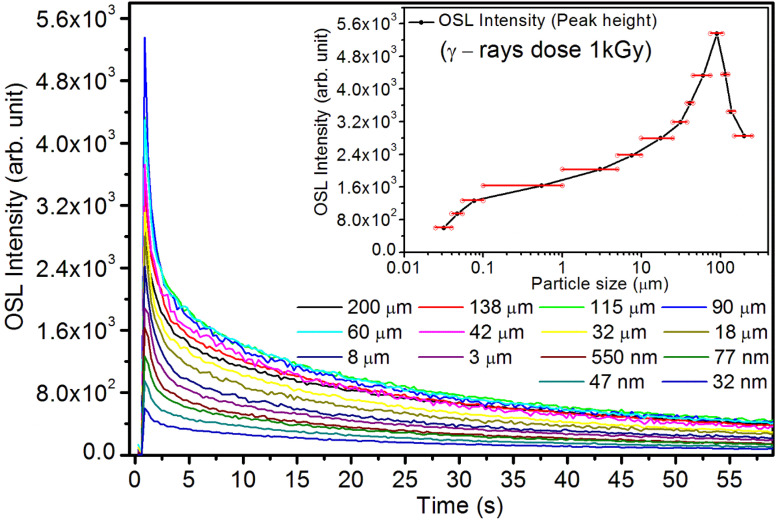
Variation of the CW-OSL intensity with time for different particle size ranges. The material was irradiated to 1.0 kGy dose of γ-rays from ^60^Co radioactive source. The variation of the intensity with the average particle size is also shown in the inset. The horizontal bars show the particle size range. The shape of the curve (intensity *vs.* particle size) shows an exponential decrease in intensity with decreasing particle size. However, for bigger particles there is decrease in the intensity as in the case of nanoparticles but for different reasons. For microcrystalline particles of bigger sizes, the stimulating light more scattered, decreasing the number of stimulating photons and the resulting CW-OSL output, while in the case of nanoparticles, it is the particle size effect.

**Fig. 6:**
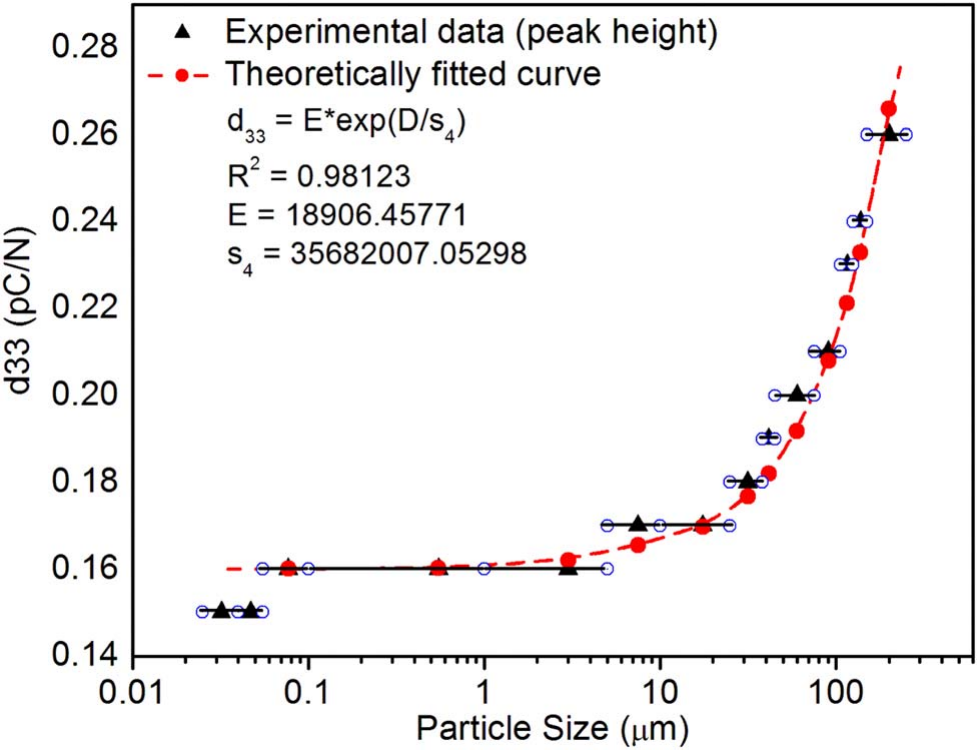
Variation of piezoelectric constant (*d*_33_) with the particle size of the SAOE ML phosphor material. The horizontal bars show different particle size ranges. The data has also been fitted theoretically to see the trend and it is shown in the inset. It is observed that there is a very good correlation between the piezoelectric constant and the particle size. It could be seen that the piezoelectric constant decreases exponentially with the particle size decreasing. The formulation used for fitting is also given in the inset.

**Fig. 7:**
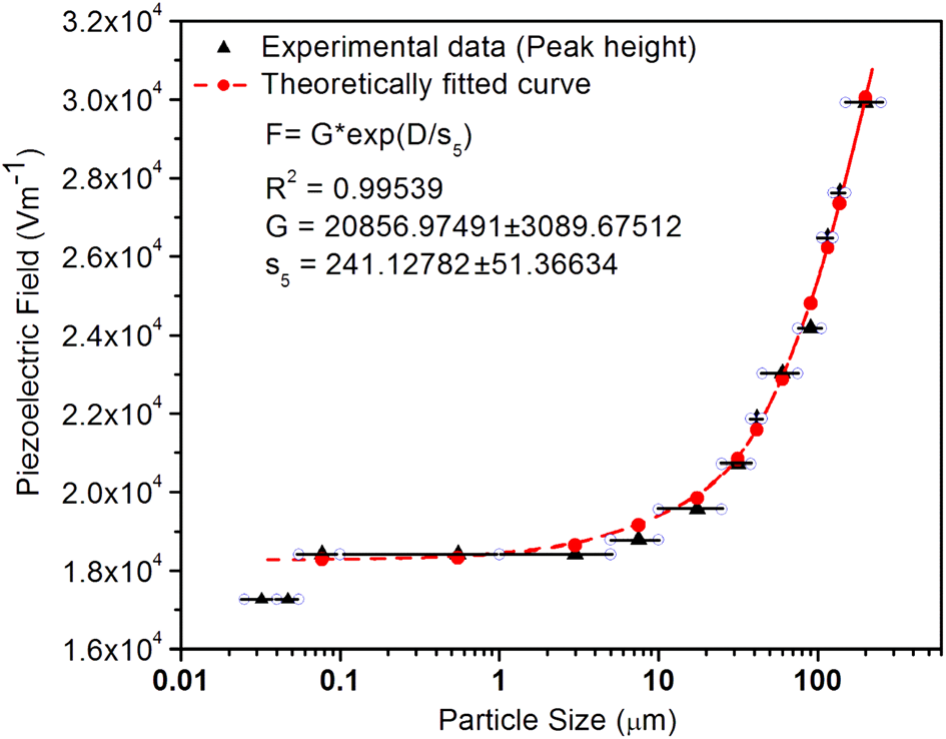
Variation of piezoelectric field with the particle size of the SAOE ML phosphor material. The data has also been fitted theoretically to see the trend and it is shown in the inset. It is observed that there is a very good correlation between the piezoelectric field and the particle size. It could be seen that the piezoelectric constant decreases exponentially with the particle size decreasing. The formulation used for fitting is also given in the inset.

**Fig. 8:**
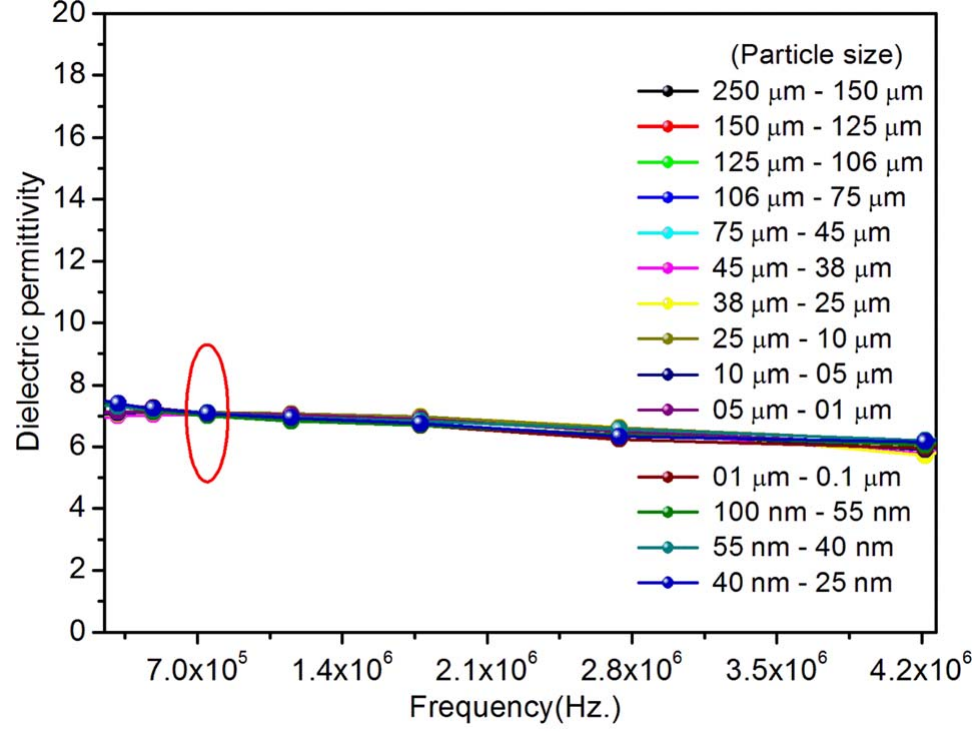
The dielectric constant measurements of the SAOE phosphor of different particle sizes. It could be seen in the figure that the dielectric permittivity of the materials is constant at low frequencies (∼0.7 MHz).

It may be understood that the defects/dislocations introduced during crushing/ball milling would be different than those produced during synthesis and as ML is the combined effect of dislocation movements and the traps, the decrease in ML intensity with particle size is as expected. To the best of our knowledge, such systematic ML studies with varying particle sizes (from micro- to nanocrystalline range) are not available in the literature and hence the explanation for such a behavior. All the materials were given proper heat treatment (annealed at around 200 °C and quenched to room temperature before taking ML). To see whether such behavior is seen in other similar luminescence phenomena, their TL and OSL were taken without irradiation and after irradiating with different doses of γ-rays from ^60^Co radioactive and UV radiation sources. The TL glow curves and OSL decay curves of nonirradiated samples of different particle size ranges (not given here due to paucity of space) are found to be similar to that of the irradiated samples. The TL and OSL results of the irradiated samples are shown in Fig. 4 and 5. It was thought that as the unirradiated phosphor material could give ML, as the traps and luminescence centers (LCs) must already be available inside. Therefore, TL and OSL of the unirradiated samples were also taken. OSL was very weak and not given here, but TL results are given in Fig. 12 below (in Section 3.7). It could be seen in these figures that the TL and OSL intensity is also showing the same trends, *i.e.*, the decreasing intensity with the decreasing particle size. It can also be seen in Fig. 4 that there is a shift (∼25 K) to the higher side in the peak temperatures of nanocrystalline materials. This is as expected as there would be a widening of the band gap of the nanocrystalline materials due to particle size effect and so the subsequent increase in trap levels of the doped impurity/activation energies. In the present case, the activation energies of the irradiated micro- and nanocrystalline materials were determined using Chen's semiemperical formulae^92^ given in Section 3.6 and are found to be 0.74 and 1.24 eV for the two TL peaks appearing at around 336 and 435 K, respectively. The decrease in intensities with particle size in the case of TL and OSL phosphors have been reported and discussed in our earlier papers.^93–96^ Accordingly, the variation in TL peak temperatures for the different particle size ranges may be attributed to strain/stress developed during crushing/ball milling and may also be due to the interactions of surface defects (Fs-centers, which are surface analogues of volume F-centers) with the electron traps developed deep inside (volume F-centers).^97^ However, in the case of ML, as it is related to the movement of dislocations and also their interactions with the traps,^80^ the decrease in intensity with particle size could not be explained on the basis of different traps related to the impurity doped. Firstly, the length of the dislocations would be shorter in the case of nanoparticles as compared to that in the case of the particles in the micro-range and hence the number of such traps in a particle would be less though such number of particles per unit volume would be more. However, as discussed earlier, local electric field around the twin interface due to twinning pseudo-elastic deformation under a load would be very less and may not be strong enough to release a greater number of trapped electrons. Also, in the case of nanoparticles, the movement of dislocations during deformation would be less, and the energy of interaction may not be enough to excite the hole traps to release holes to the valence band. These could be some of the reasons that the intensity is decreasing with the particle size. Further, piezoelectric constant (*d*_33_) measurements have been taken and polarization voltages on deformation were determined using the formula 
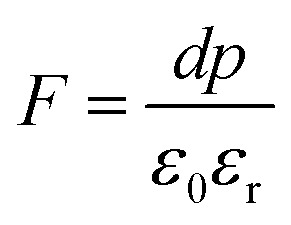
^98^ where, *F* = electric field V m^−1^, *ε*_0_ = 8.85 × 10^−12^ C^2^ N m^−2^, *ε*_r_ = 7.0 (determined by dielectric measurements), *d* = *d*_33_ (piezoelectric constant along the direction of the applied pressure) C/N, *P* = threshold load (N m^−2^). The results, *i.e.*, variation of *d*_33_ with particle size and variation of electric field with particle size, are as shown in Fig. 6 and the variation of the piezoelectric field also shows a similar trend (Fig. 7). It was also experimentally seen and ensured that the dielectric constant remains constant for low frequencies (Fig. 8).

Chandra, B. P. has given theoretical formulation^10^ for the elastico-ML for SrAl_2_O_4_:Eu^2+^ phosphor. According to him, in the presence of the piezoelectric field, the detrapping of charge carriers takes place either due to the thermal ionization of traps owing to the reduction in trap-depth caused by the piezoelectric field or due to the tunneling process. If, *P* = *P*_m_, at *t* = *t*_m_ (time required to reach the ML intensity at *I*_m_ for *P*_m_ ≫ *P*_th_ (the threshold pressure). The maximum ML intensity *I*_m (compressive ML)_ due to such a process is given by,

1 *I*_m (compressive ML)_ = *η σ* Ω *N*_C_*N*_L_*N*_t_*n*_h_*Z τ μ B*^2^*d*_0_^2^*Ṗ P*_m_

where *η* = efficiency of radiative decay of the excited state of the impurity ions (Eu^2+^/Eu^3+^) inside the phosphor material, *σ* = the capture-cross section of the energy state for the excited Eu^2+^ ions, Ω = activation volume near an effective defect centre, *N*_C_ = total number of crystallites in the sample, *N*_L_ = average number of defect centers responsible for the local piezo-electrification in the crystallites, *N*_t_ = concentration of the filled electron traps, *n*_h_ = concentration of hole centers. *n*_0_ = concentration of number of detrapped electrons caused by the piezoelectric field, *Z* = 1/*F*_c_ and *F*_c_ is the characteristic piezoelectric field, *τ* = lifetime of electrons in the conduction band, *μ* = mobility of electrons in the crystallites, *d*_0_ = is the local piezoelectric constant near the effective defect centers, *B* = correlating factor between the piezoelectric field *F*_c_ and the piezoelectric charge *Q*, *P* = maximum applied pressure, *Ṗ* = fixed pressing rate.

This is for crystallites of any size and shape. As the particle size increases or decreases, some of the quantities/parameters would change and most of the other quantities are characteristics of the material and will remain unchanged. For example, as the particle size decreases, *N*_C_, the total number of crystallites will increase in the sample; however, the average number of defects in a crystallite will decrease as the volume decreases and the number of surface defects responsible for quenching would increase due to the increase in surface to volume ratio. However, the product of *N*_C_ and *N*_L_ may remain constant. All the other parameters, *η*, *σ*, Ω, *N*_t_, *n*_h_, *Z*, *τ*, *μ*, *B*^2^, *Ṗ* and *P*_m_ are constant except *d*_0_ or *d*_33_ (piezoelectric constant along the applied pressure, *i.e.*, along *z*-direction). It can be seen in Fig. 3 and 6 that the variation of ML intensity with the particle size and also the variation of *d*_33_ values and variation of calculated values of the piezoelectric field F have the same exponentially decreasing trends, *i.e.*, *d*_33_ = *A* × exp(*D*/*s*_1_), where *A* = 18.91 × 10^3^, *s*_4_ = 35.68 × 10^6^, *D* (crystallite size) varies from 20 nm to 210 μm, and *d*_33_ varies from 0.15–2.8 pC/*N*. If we put this expression in eqn (1), and consider all the other parameters constant, then the variation in ML intensity with *d*_33_ would be exponential, *i.e.*,

2 *I*_(compressive ML)_ (*d*_33_) = *η σ* Ω *N*_C_*N*_L_*N*_t_*n*_h_*Z τ μ B*^2^*Ṗ P*_m_*A*^2^ exp (2*D*/*s*_4_)

where, *A* is a constant and *s*_4_ is another correlation constant.

Now, we can see that the ML intensity varies exponentially with *d*_33_. Again, as mentioned earlier, there is a linear relationship with *d*_33_ and with the piezoelectric field, *i.e.*, *F* = *dp*/*ε*_0_*ε*_*r*_, and the variation of the intensity with the piezoelectric field would be

3 *I*_(compressive ML)_ (*F*) = *η σ* Ω *N*_C_*N*_L_*N*_t_*n*_h_*Z τ μ B*^2^*Ṗ P*_m_*C*^2^ exp (2*D ε*_0_*ε*_r_/*p s*_5_)

where *C* is another constant and *s*_5_ is another correlation constant.

From the above equation, *p*_m_ (maximum pressure), *ε*_0_, permittivity in vacuum, *ε*_r_, relative permittivity or dielectric constant and *s*_5_ are constants; therefore, the intensity would vary exponentially with the particle size. Our experimental results (Fig. 3, 6, and 7) support these theoretical formulations.

### 3.6 Effect of particle size on ML decay curves and intensity by impulsive measurements

**Fig. 9:**
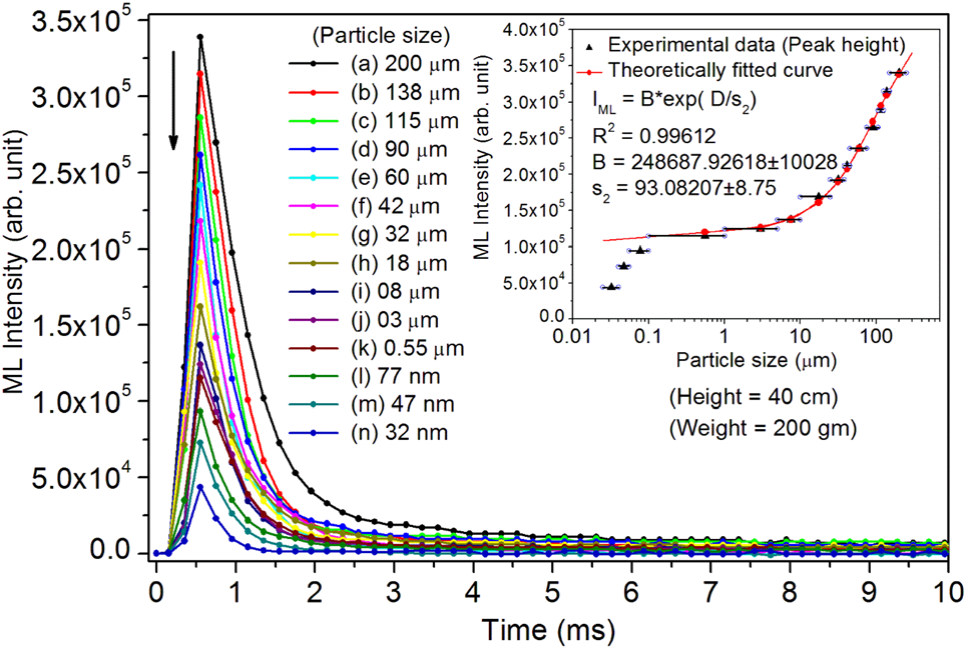
Variation of the ML (impulsive) intensity with time for different particle size ranges. The variation of the intensity with the average particle size is also shown in the inset. The horizontal bars show the particle size range. The curve (intensity *vs.* particle size) has also been theoretically fitted and shows an exponential decrease in intensity with particle size decreasing. The equation with different parameters is also given in the inset. There is a very good fit up to the submicron size range. However, at the nanoparticle size range, a slight deviation could be seen here also.

As mentioned earlier in the Experimental section, the ML measurements were also made using another ‘*home-made*’ setup where a piston (a solid cylindrical rod) of certain mass is suddenly dropped through a pipe onto the sample material using a pulley and stainless-steel wire. In this experiment, particles in different particle size ranges were used to study the effect of particle size on the ML decay curves and their intensity, keeping the height and the velocity of the piston constant. The experiments were done by dropping the mass from different heights. However, the results only for a mass and typical height (200 g and 40 cm, respectively) are given here (Fig. 9). We have tried to fit the data theoretically, and the fitted curve and the formulation have also been shown in the inset of the figure.

According to Sharma *et al.*,^32^ a crystallite (particles) breaks into other particles with smaller and smaller particles with increasing strain. This would be possible for bigger (microcrystalline) particles. However, for nanocrystalline materials, it is unlikely that nanosized crystallite would break into smaller particles by dropping a small mass on the material spread over a transparent base from a low height.^10^ In such circumstances, it may be considered that the impact stress remains in the elastic region. If *f* is the fraction of maximum elastic energy and the rigid impacting object of mass *m* dropped from a height *h* with the velocity *v*, *σ*_0_ is the maximum value of the impact stress and the elastic energy imparted with the powder material of total volume *V* and Young's modulus of elasticity *Y*, then the maximum ML intensity is given by,^10^4
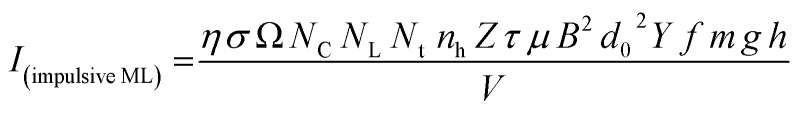
where *g* is acceleration due to gravity. Now, as mentioned earlier, putting the value of the piezoelectric constant in terms of *D*, *d*_33_ = *A* exp(*D*/*s*_4_) in the above equation, we get,5
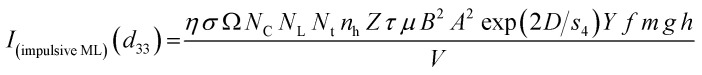
where, *A* is a constant and *s*_4_ is another correlation constant.

Here, we could see that as the *d*_33_ is varying exponentially with particle size (Fig. 6), the ML intensity would also be varying exponentially with the particle size during impulsive measurements. Also, as Young's modulus of elasticity is less for nanoparticles as compared to that of the bulk,^99,100^ the ML intensity would also be low.

It could be seen from the above equation that the ML intensity in terms of the piezoelectric field is varying exponentially with the particle size (Fig. 7) as all other parameters are constant and in terms of the piezoelectric field again it would be6
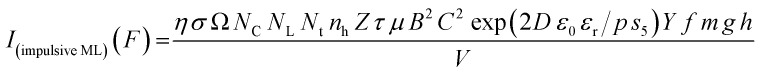
where, *C* is a constant and *s*_5_ is another correlation constant.

Here, there is a linear relationship of *F* with *d*_33_, *i.e.*, *F* = *d*_33_*p*/*ε*_0_*ε*_r_. In terms of piezoelectric field, the ML intensity would be varying exponentially with the particle size during the impulsive measurements (Fig. 9).

### 3.7 Redox reactions and their effect on the luminescence characteristics of SAOE phosphor

**Fig. 10:**
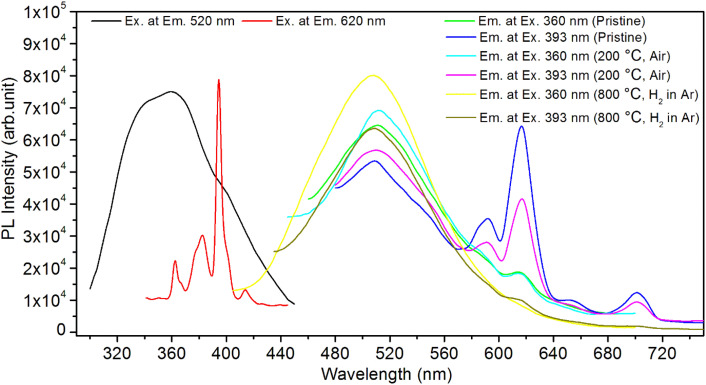
PL excitation and emission spectra of SAOE phosphor annealed in oxidizing (air) and reducing (10% H_2_ in Ar) atmospheres at different temperatures. The spectra for the pristine (as prepared) sample are also shown in the figure. The average particle size was in the range of (75–106 μm). Approximately the same amount of the sample material was taken every time to record the spectra. PL study was also done for the nanocrystalline materials but not given here due to the paucity of space and similarity in the spectra except for the low intensity and slight shifts in their peak positions.

**Fig. 11:**
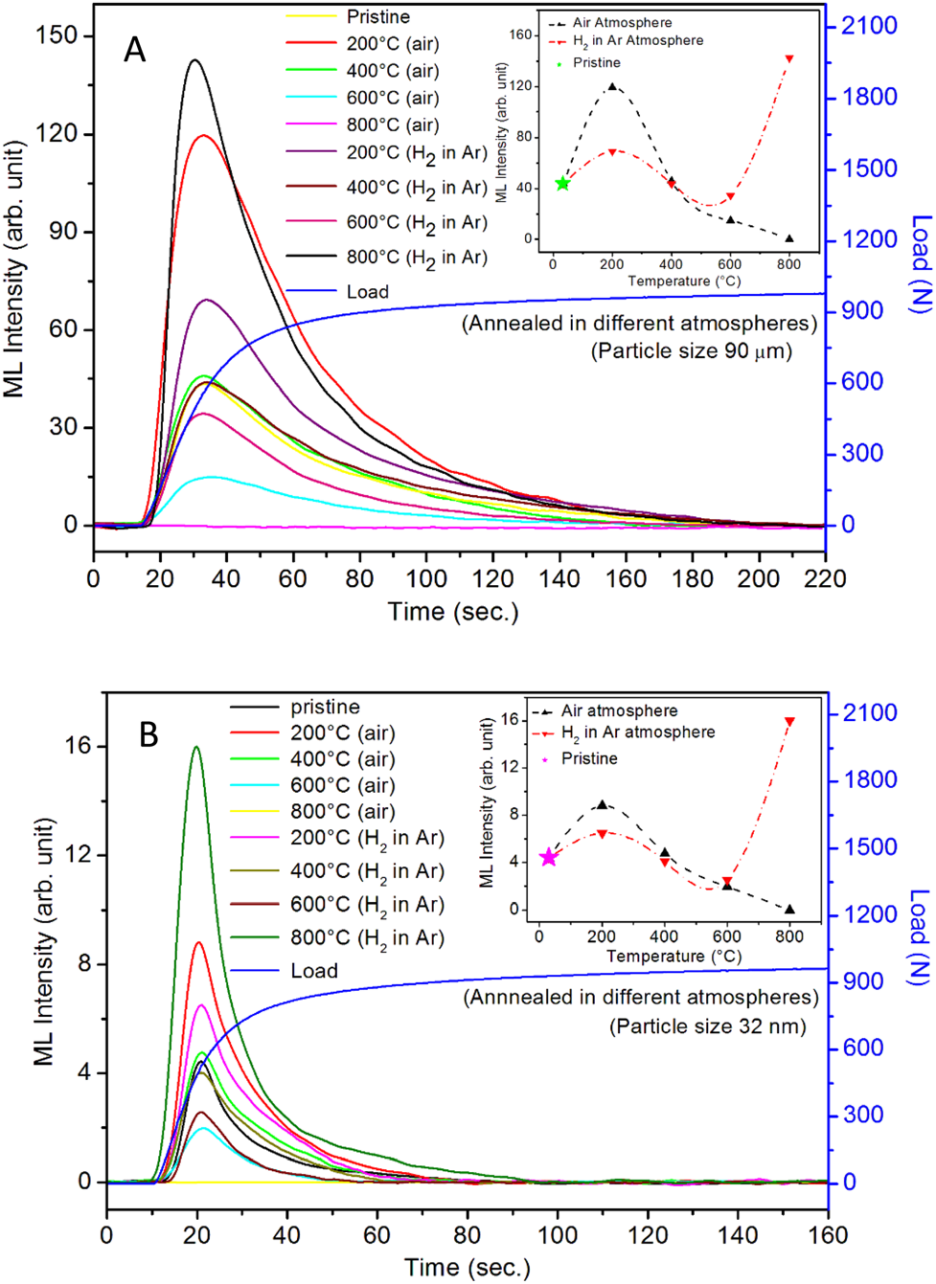
(A) The effect of annealing temperature after annealing in oxidizing (air) and reducing (10% H_2_ in Ar) atmospheres on the microcrystalline SAOE phosphor. The ML of pristine (as prepared) material is also shown for comparison, (1) as prepared, (2) annealed in air at 200 °C, (3) air at 400 °C, (4) air at 600 °C, (5) air at 800 °C, (6) annealed in reducing (10% H_2_ in Ar) atmosphere at 200 °C, (7) (10% H_2_ in Ar) at 400 °C, (8) (10% H_2_ in Ar) at 600 °C, (9) (10% H_2_ in Ar) at 800 °C. The variation of the maximum ML intensity with particle size is also shown in the inset. The particle size was in the range of 106–75 μm. (B) The effect of annealing temperature after annealing in oxidizing (air) and reducing (10% H_2_ in Ar) atmospheres on the nanocrystalline SAOE phosphor. The ML of pristine (as prepared) material is also shown for comparison, (1) as prepared, (2) annealed in air at 200 °C, (3) air at 400 °C, (4) air at 600 °C, (5) air at 800 °C, (6) annealed in reducing (10% H_2_ in Ar) atmosphere at 200 °C, (7) (10% H_2_ in Ar) at 400 °C, (8) (10% H_2_ in Ar) at 600 °C, (9) (10% H_2_ in Ar) at 800 °C. The variation of the maximum ML intensity with particle size is also shown in the inset. The particle size was 32 nm.

**Fig. 12:**
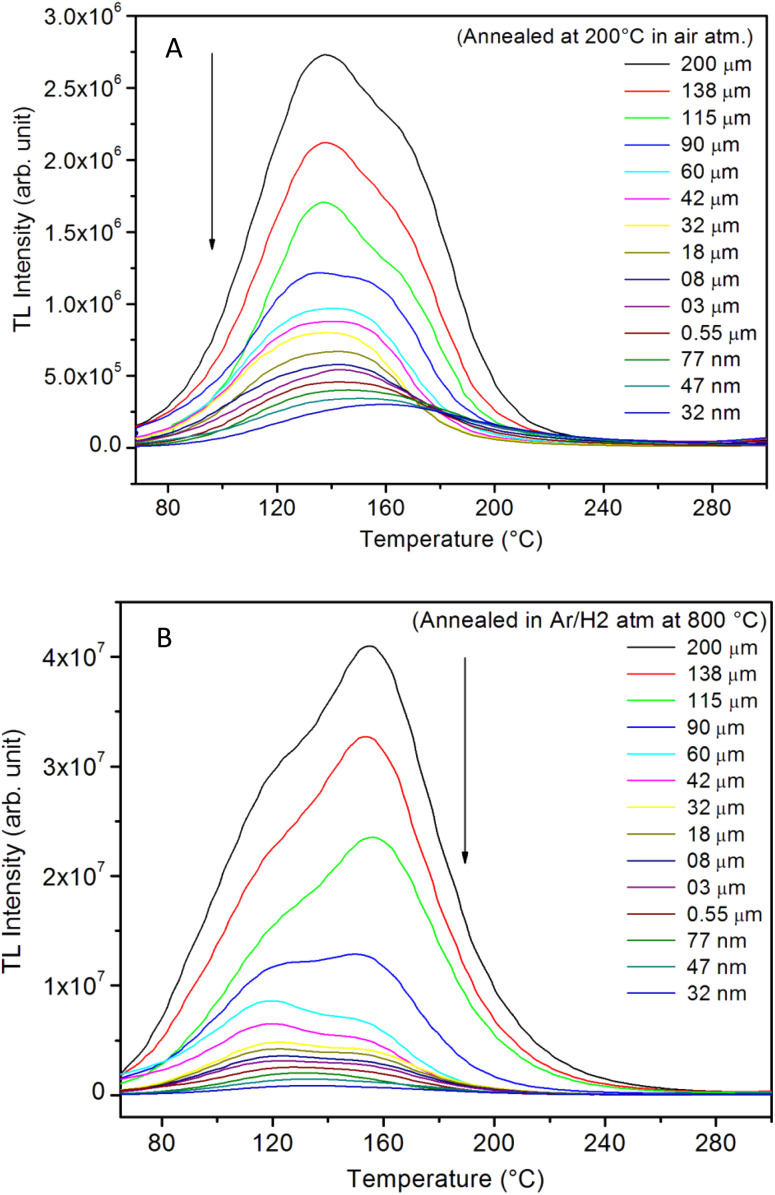
Effect of the annealing at the optimized temperatures, *i.e.*, (200 °C) in oxidizing (air) and (800 °C) in reducing (10% H_2_ in Ar) atmospheres on TL glow curves for different micro- and nanocrystalline particle sizes of the unirradiated SAOE ML phosphor: (A) annealed in air at 200 °C, (B) annealed in 10% H_2_ in Ar atmosphere. The arrows pointing downwards show a decrease in TL intensity with the particle size decreasing. It could also be noted that the intensity of each particle size increases more than ten times on annealing in reducing (10% H_2_ in Ar) atmosphere.

It is known that even if we dope a phosphor material initially using an impurity salt consisting of the ions in a particular ionic state, it is not necessary that they would be incorporated inside the matrix in the same form, and they may get incorporated in different ionic states (if exists naturally).^101–103^ For example, in the present study for doping Eu^3+^ ions in a phosphor material, we used Eu(NO_3_)_3_ salt consisting of Eu^3+^ ions, and it could be seen in the PL spectra (Fig. 10), they have entered in both forms, Eu^3+^ as well as Eu^2+^. It is also known that Eu^2+^ ions give better sensitivity due to their emission in the blue-green region; therefore, to enhance the sensitivity, the phosphor material is annealed in a reducing atmosphere to convert most of the Eu^3+^ ions into Eu^2+^ ions and also protects Eu^2+^ ions from oxidizing into Eu^3+^ ions on annealing in air. It is important from an application point of view; if we need to compromise with the intensity for particle size, then there should be some method to improve the intensity. Therefore, to see the effect of annealing in different atmospheres at different temperatures, the ML phosphor materials under investigation (of all particle sizes) were annealed in oxidizing (air) and reducing (10% H_2_ in Ar) atmospheres at different temperatures for 1.0 h to optimize the annealing temperature for their ML, TL, and PL studies. It can be seen in Fig. 11A that the phosphor (average particle size ∼90 μm) shows maximum ML intensity at around 200 °C in oxidizing (air) and 800 °C in reducing (10% H_2_ in Ar) atmosphere. This was done in the case of nanomaterials also for comparison and the typical ML results (for 32 nm particle size) are given in Fig. 11B. The ML, TL, and PL of all the materials annealed at the optimized temperatures, *i.e.*, annealed at 200 °C in air and at 800 °C in a reducing atmosphere, were done. However, the results for only selected particle sizes are shown here due to the paucity of space and the similarity of the results. Results on PL are shown in Fig. 10 while the TL results are in Fig. 12. The results for pristine (as prepared material) are also shown here for comparison. It can be clearly seen in Fig. 11(A and B) that the ML peak intensity of the material annealed in the reducing atmosphere increases with temperature and is always more than that of the pristine material, while that of the material annealed in oxidizing atmosphere first increases up to 200 °C and then decreases on annealing at further higher temperatures and completely diminishes at around 800 °C. To understand the phenomenon, we studied the excitation and emission PL spectra of these materials. It could be observed from PL results (Fig. 10) that the excitation spectra for the emission peak at around 620 nm is quite different than that for emission at around 510 nm. Firstly, the former spectrum has very sharp bands than the latter one and secondly, the prominent peak is at around 393 nm while that in the latter one is at around 360 nm. Generally, the former spectrum (especially the 393 peak) is considered for the excitation of Eu^3+^ ion species and the latter one (the 360 nm band) is for Eu^2+^ ions. The broad band at around 510 nm is considered to be due to 4f^6^5d^1^ → 4f^7^ transitions of Eu^2+^, while the emissions at around 620 nm are considered to be as charge transfer band (CTB) between Eu^3+^ and O^2−^ ions. This band could be attributed to the forced electric dipole transition ^5^D_0_–^7^F_2_ of Eu^3+^ ions, while a group of emissions peaking at lower (centered around 590 nm) as well as at higher wavelengths (centered around 700 nm) are from ^5^D_0_–^7^F_*j*_ (*j* = 0, 1, 2, 3, 4) and are well-known.^104–107^ It is also known that Eu gets incorporated in strontium aluminum oxide (SAO) matrix in the form of Eu^2+^ as well as Eu^3+^. This is very much evident from the excitation/emission spectra given in Fig. 10. The annealing in oxidizing and reducing atmospheres at different temperatures decrease or increase the relative number of such ion species due to redox reactions and these are very important to improve the luminescence intensity of the phosphor and in the present case, the ML intensity of the SAOE phosphor is found to be highest by converting most of the Eu^3+^ to Eu^2+^ (as evident from emission spectrum) on annealing in reducing atmosphere at 800 °C.

**Fig. 13:**
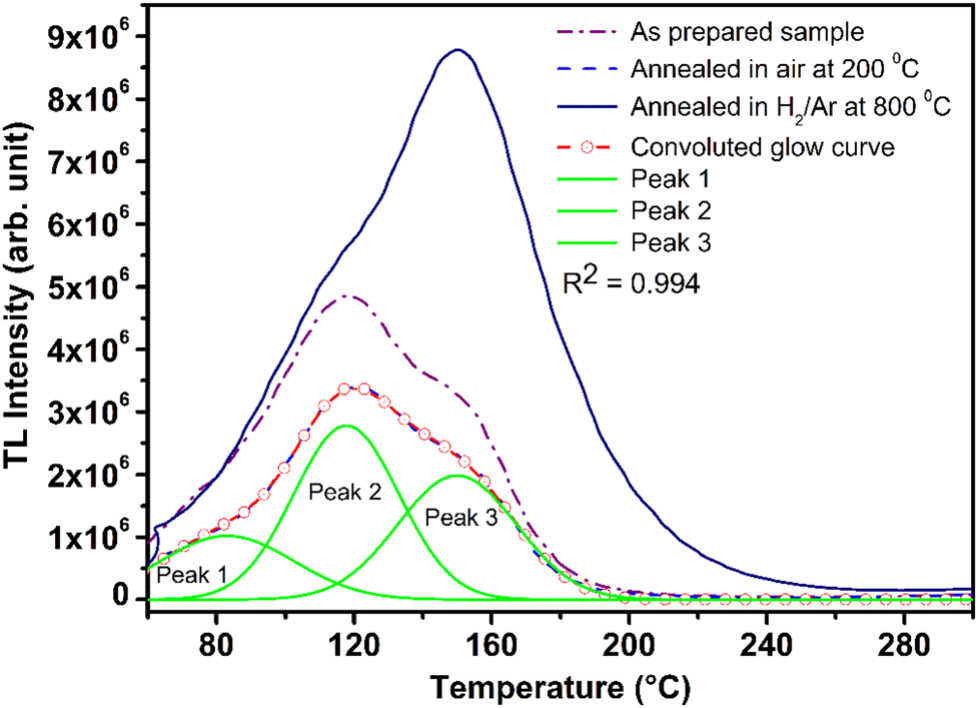
Deconvolution of a TL glow curve by peak shape method using Kitis’ formula for second order kinetics. The TL glow curves of the pristine (as prepared) and the materials annealed at the optimized temperatures, 200 °C in air and 10% H_2_ in Ar atmospheres are also shown. Three TL peaks exist in the glow curves and deconvoluted successfully, shown as Peak 1, Peak 2, and Peak 3 in the figure. Trapping parameters, including trap depth have also been determined using these peaks and is given in Table 1.

**Fig. 14:**
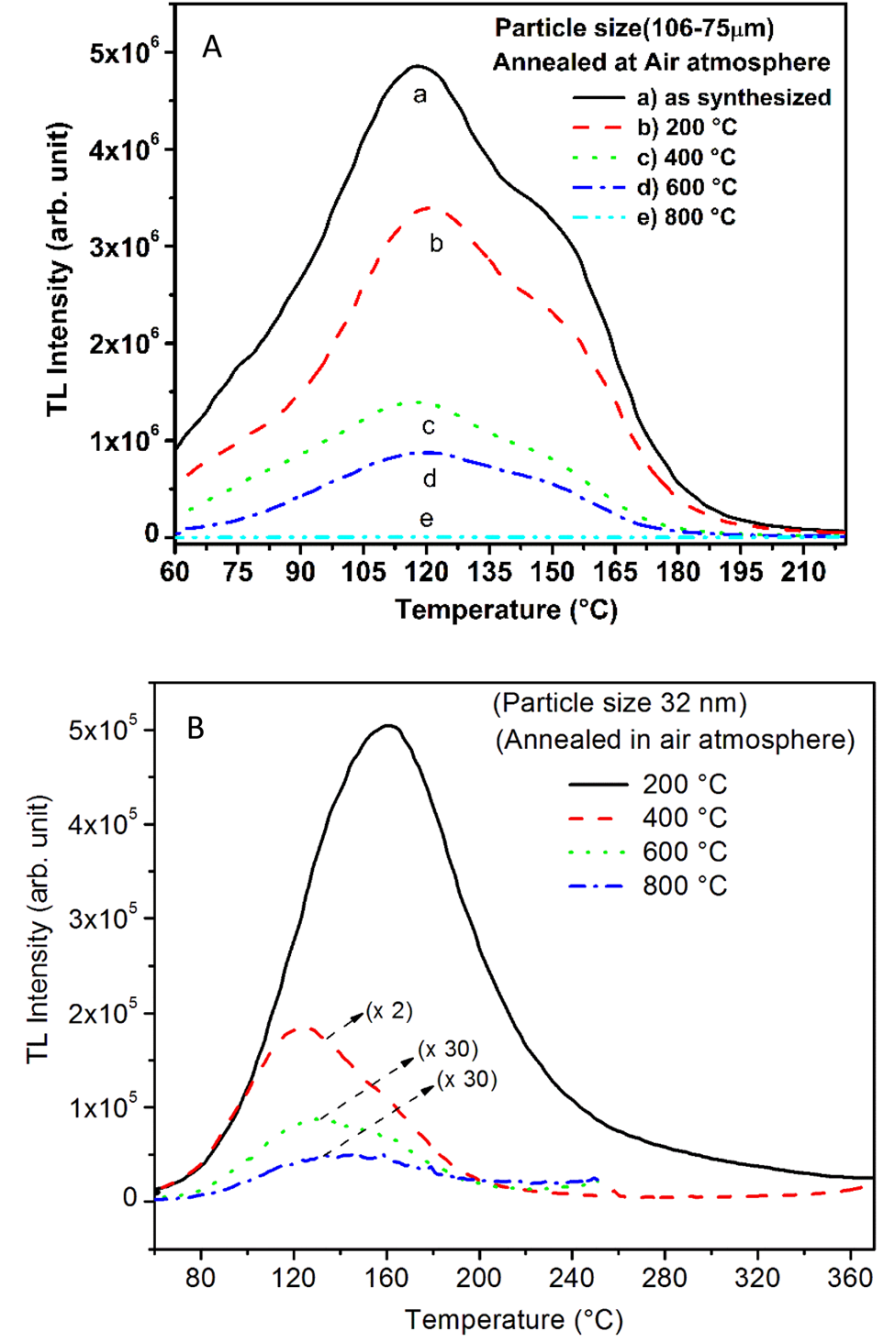
TL glow curves of unirradiated SAOE phosphor materials annealed at different temperatures in air atmosphere, (A) microcrystalline material (106–75 μm) and (B) nanocrystalline material having particle size ∼32 nm. Decrease in the TL intensity with particle size without any change in the glow curve structure of the microcrystalline material could be clearly seen in the figure. However, in the case of nanomaterial a shift in the peak temperature(s) could also be seen with the annealing temperatures. The TL glow curves of the nanocrystalline material annealed in the temperature range of 400–800 °C have been multiplied by the factors near these curves and the ordinate (*Y*-axis) scale needs to be divided these factors to see the actual intensity of these curves.

**Fig. 15:**
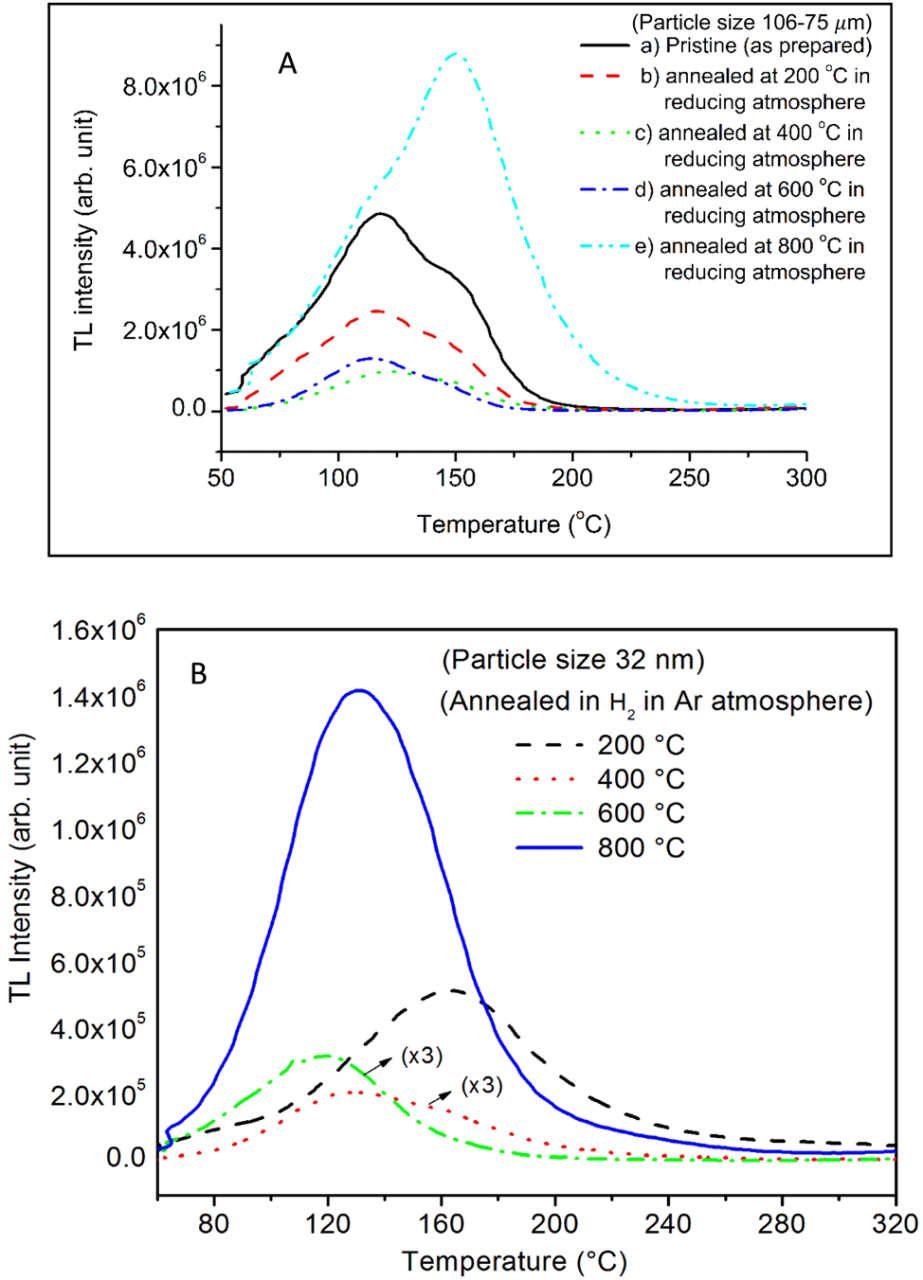
TL glow curves of SAOE phosphor materials annealed at different temperatures in reducing (10% H_2_ in Ar) atmosphere, (A) microcrystalline material (106–75 μm) and (B) nanocrystalline material having particle size ∼32 nm. Decrease in the TL intensity with particle size without any change in the glow curve structure of the microcrystalline material could be clearly seen in the figure. However, in case of nanomaterial a shift in the peak temperature(s) could also be seen with the annealing temperatures. The TL glow curves of the nanocrystalline material annealed in the temperatures 400 and 600 °C have been multiplied by the factors near these curves and the ordinate (*Y*-axis) scale needs to be divided these factors to see the actual intensity of these curves.

Further, to investigate the kind of traps and their activation energies (trap depths) TL (without any irradiation) of the materials (pristine, annealed in reducing atmosphere and oxidizing atmosphere) was taken. The results are given in Fig. 12. As expected, the TL intensity of the material annealed in the reducing atmosphere (10% H_2_ in Ar) was found to be high and that of the material annealed in the oxidizing atmosphere is low as compared to that of the pristine (as prepared) SAOE phosphor material. This was expected because the luminescence is due to traps related to Eu^2+^ and Eu^3+^ ions and as the relative number of these impurity ions vary, the intensity would vary. These studies have been done for micro- as well as nanocrystalline materials. Typical representative glow curves at optimized annealing temperatures of 200 °C for annealing in oxidizing atmosphere (air) and 800 °C for reducing atmosphere (10% H_2_ in Ar) are given in Fig. 12(A and B). It could be seen in these figures that the intensity goes on decreasing with the particle size. These results are similar to our earlier results.^36^ It is known that the TL sensitivity is poor in case of nanophosphors due to surface to volume ratios and different kinds of surface and volume defects. Especially, the unsaturated dangling bonds and other surface defects could act as quenchers. Moreover, traps near the surface may get destroyed by the adsorption of atmospheric water molecules. There were some changes in the glow curves and in relative intensities of their peaks. The changes in the peak temperatures may be because of the widening of the band gap with decreasing particle size and the relative changes in the trap depths (activation energies), while the changes in the sensitivities could be attributed to the changes in relative number of electron traps at different localized energy levels related to impurities of different ionic states, *i.e.*, Eu^2+^ and Eu^3+^ and probabilities of recombining with hole traps/luminescence centers (LCs) and/or retrapping. This is also in line with that of the ML and PL results. There are three peaks observed in TL glow curves of the unirradiated sample at around peak temperatures 357, 389, and 422 K, peaks 1–3, respectively (Fig. 13). The glow curve was deconvoluted using Kitis' formula (eqn (7) given in Section 3.8)^108^ for second order kinetics as the form factor was found to be around ∼0.5 for all the three peaks. Further, the activation energies for traps responsible for these peaks (peak 1–3) were found to be 0.72, 1.14, and 1.28 eV, respectively. The TL was recorded for the SAOE phosphor materials annealed in an oxidizing atmosphere (air) at different temperatures. The results are shown in Fig. 14(a and b). The overall TL intensity of these materials is found to decrease with the increasing annealing temperature. This was expected as a greater number of Eu^2+^ ions get oxidized to Eu^3+^, the TL for microcrystalline (Fig. 14a) as well as for the nanophosphor (Fig. 14b) goes on decreasing and finally diminishes at around 800 °C. These results are similar to the results for the EML shown earlier in Fig. 3. Similar study was also done for the materials annealed in a reducing atmosphere. The TL results for micro- and nanocrystalline materials are shown in Fig. 15(A and B). It could be observed in these figures that the TL intensity of the material annealed at around 200 and 400 °C decreases with temperatures increasing though a greater number of the Eu^3+^ ions are getting reduced to Eu^2+^ ions, while the ML intensity goes on increasing. This could be explained as follows. As the phenomena of ML is dependent on both the movements of dislocations and the traps and the local electric field developed between the twining boundaries, such movements would be more while applying the loads, and as the number of Eu^2+^ ions have increased after annealing, the combined effect would be more predominant. However, TL is stimulated only thermally; there could be more nonradiative recombinations of the shallow traps leading to early fading. Also, as it follows second order, there would also be more retrapping and the TL yield be less. Also, there is phase transition from monoclinic to hexagonal phase in the temperature range of 600–800 °C.^81^ This would lead to a greater number of dislocations (strain/stress) and traps and their retention after annealing and sudden quenching to room temperature. Therefore, it is expected that the stress/strain would be released during heating while TL readouts are taken, leading to more movements of the dislocations and faster release and recombinations of the traps. The combined effect would result in enhancement of TL. Thus, TL as well as ML would go on increasing for the samples annealed above 600 °C in reducing atmosphere.

### 3.8 Trapping parameters and activation energy (trap depth)

Typical TL glow curves of all the micro- and nanocrystalline materials annealed at the optimized temperatures, *i.e.*, at around 200 °C in oxidizing (air) and 800 °C in reducing (10% H_2_ in Ar) and that of the pristine (as prepared) are shown in Fig. 12. All the glow curves look alike. Therefore, one of the curves (TL glow curve for the material annealed at 200 °C and without any irradiation was deconvoluted (Fig. 13) to separate individual glow peaks to determine their depths. Kitis' formula for second order kinetics was used as the value of *μ*_g_ is ∼0.52.^108^7
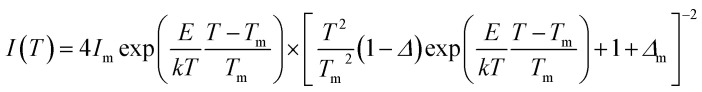
with, *Δ* = 2*kT*/*E*, *Δ*_m_ = (2*kT*_m_)/*E* and *I*(*T*) is the glow peak intensity at temperature *T*, *E*_a_ (eV) is the activation energy, *k* (eV K^−1^) is the Boltzmann's constant, and *T*_*m*_(*K*) is the peak position in *K*. *E* is the thermal energy, needed to eject electrons from the trap centers, calculated using the following set of equations (Chen's formulae),^93^ which are independent of the order of kinetics:8
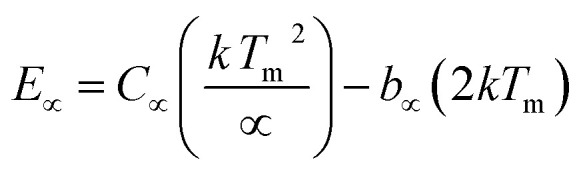
with, *α* = *τ*, *δ*, *ω*, where, *τ* = *T*_m_ − *T*_1_, *δ* = *T*_2_ − *T*_m_, *ω* = *T*_2_ − *T*_1_, *μ*_g_ = *δ*/*ω*, *c*_τ_ = 1.51 + 3.0 (*μ*_g_−0.42); *c*_δ_ = 0.976 + 7.3 (*μ*_g_−0.42); *c*_ω_ = 2.52 + 10.2(*μ*_g_ − 0.42); *b_τ_* = 1.58 + 4.2(*μ*_g_ − 0.42); *b*_δ_ = 0; *b*_ω_ = 1.

The trapping parameters, including trap depths (activation energies), are given in Table 1.

**Table 1:** Trapping parameters of SrAl_2_O_4_:Eu material annealed in an oxidizing atmosphere (air) for 1 h. The material was not given any radiation dose. Here, *μ*_g_ is form factor ∼0.52 for second order, b is order of kinetics, and *E*_a_ is activation energy required for releasing the trapped electron(s)

Peak	*T*_1_ (K)	*T*_m_ (K)	*T*_2_ (K)	*ω* = *T*_2_ − *T*_1_ (K)	*τ* = *T*_m_ − *T*_1_ (K)	*δ* = *T*_2_ − *T*_m_ (K)	*μ*_g_	*b*	*E*_a_ (eV)
1	334	357	379	45	23	22	0.49	2	0.72
2	371	389	407	36	18	18	0.50	2	1.14
3	403	422	442	39	19	20	0.51	2	1.28

### 3.9 Repeated measurements of ML, TL and regeneration of traps

**Fig. 16:**
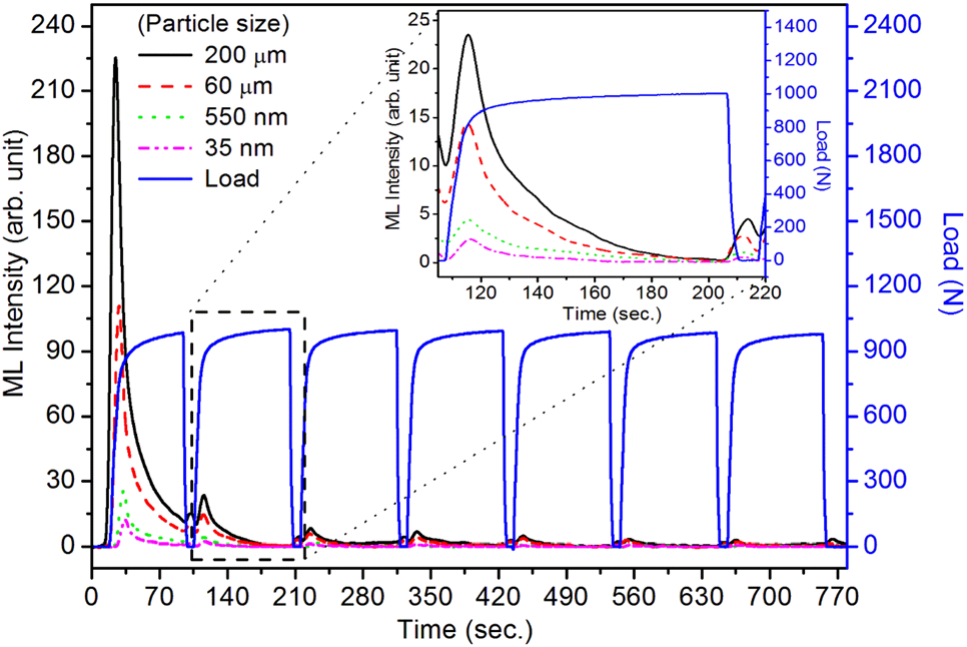
The ML glow curves of the SAOE phosphor material annealed in air at 200 °C for 1.0 h for repeated ten cycles of the load. However, the variation with seven cycles only is shown for better clarity. The enlarged view of a selected (cycle-2) has also been shown in the inset. It could be clearly seen in the figure that the maximum ML intensity is diminishing with increasing the number of repeated cycles.

The repeated measurements of ML for micro- as well as nanocrystalline materials were taken. During this process, the ML was taken repeatedly for the same material while applying the load as well as during its release. The results are given in Fig. 16. It can be seen in the figure that at the first time, the ML intensity is the highest and goes on decreasing with subsequent repeated measurements. It could also be observed in the occurrence of ML during the release of the load though its intensity is much less (∼4 times less than emitted during applying load at the first time). This could be explained on the basis of movements of dislocations and excitation of traps. While applying load, it is obvious that there would be much more movements of the dislocations and the generation of local electric fields between the twining boundaries would be higher that would stimulate a greater number of traps than during releasing the load. Further, as there are shallow (0.72 eV) and deep (1.14) and still deeper (1.28 eV, almost double) traps (Table 1), some of the shallow traps emptied while applying the load at the first time may be filled by the deep traps and their positions by much deeper traps. This could actually be observed in Fig. 16 (inset). The figure, in fact, is the enlarged view for one of the ML cycles (2^nd^ cycle). This ML glow curve has been divided into four regions: (i) in the first region, when the dynamic compressive load is applied, the ML intensity increases almost linearly with the load, (ii) when the load the almost constant, it starts decaying; however, it could be clearly seen that there are shoulders (bumps) in these decay curves (as regions II and III) while an almost constant load is applied and also get repeated in each cycle. This cannot occur without the movements of the traps. Further, this (applied load) leads to phase transition at room temperature under load.^81^ So, when the load is released at the same rate as it was applied, there would again be some movements of dislocations and excitations/recombinations of traps, leading to ML, though the intensity of this glow peak was observed to be much less than that of the same while applying load. This could be explained as follows. While applying the load the first time, certainly, the number of traps and dislocations would be at the highest, so the combined effect due to movements of the dislocations and that of excitation/release of traps near the impurity ions would be more giving rise to maximum EML intensity. Also, the effect of phase transition^95^ could not be ignored. However, during the release of the load, the number of traps/dislocations would be less, and the percentage of the material which has gone through the phase transition may not get reversed completely this time. Hence, as the combined effect, the intensity would be less. In subsequent runs, lesser and lesser intensity would be observed till all the traps are diminished. Thus, the mechanism of emptying the shallow traps and getting filled by the deeper traps seems plausible. This may continue even under load, and thus, a shoulder (bumps) during the decay of the ML was observed. Comparative study of the micro- and nanomaterials (Fig. 16) show many similarities in the results of the repeated measurements except for the low intensity during loading as well as releasing. This is also in line with the earlier ML results that the intensity is poor in the case of nanophosphors.

**Fig. 17:**
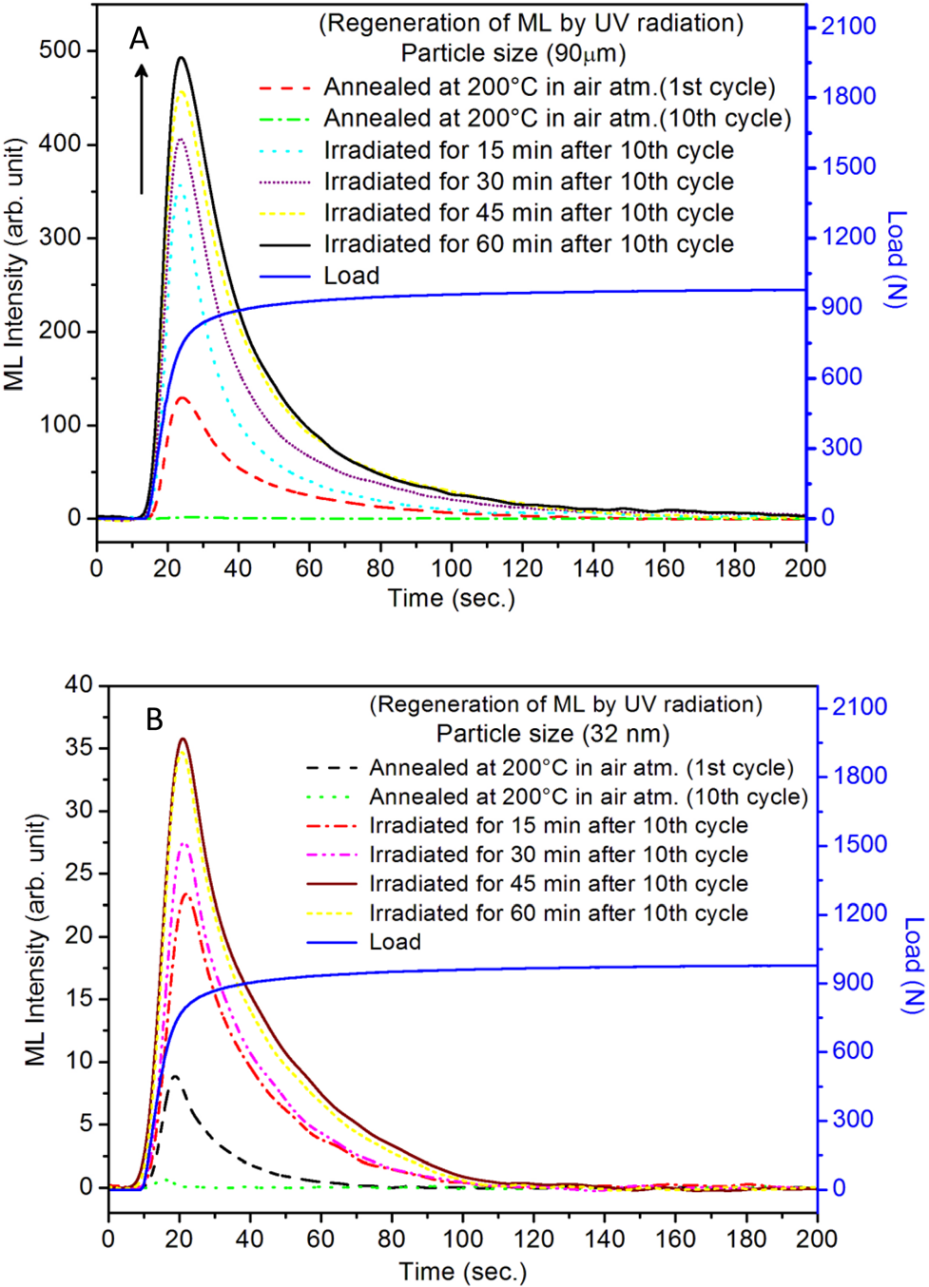
(A) Regeneration of ML of micro- (106–75 μm) and nanocrystalline (∼32 nm) by UV-irradiation after ten cycles of ML readouts: (a) the ML glow curves of the materials after ten readouts and irradiation for different UV-irradiation time periods are shown in the figure. The ML glow curves of the first cycle and that of the tenth cycle are also shown for comparison, (B) the ML glow curves of the materials after ten readouts and irradiation for different UV-irradiation time periods are shown in the figure. The ML glow curves of the first cycle and that of the tenth cycle are also shown for comparison. No ML residue was observed after the tenth cycle of the ML readout in both the cases. The increase in the ML intensity with the UV-irradiation time periods could also be observed. The only difference in these two cases is that the intensity of the nanocrystalline is low.

**Fig. 18:**
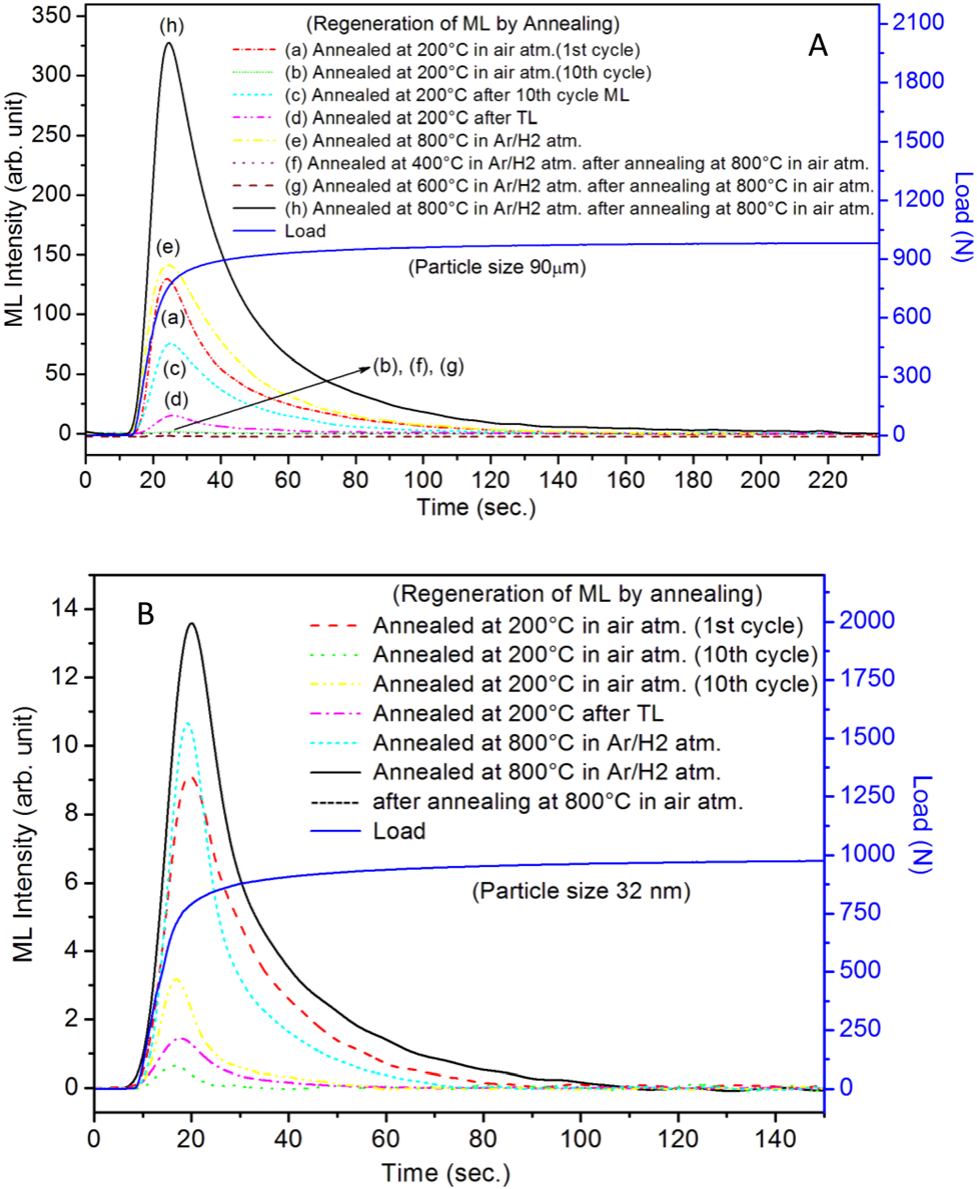
(A) Regeneration of ML of micro- (106–75 μm) and nanocrystalline (∼32) nm by annealing after ten cycles of ML readouts: (a) the ML glow curve of the material annealed at 200 °C in air for the first cycle of readout, (b) the ML glow curve of the same materials after ten cycles of readouts, (c) ML glow curves of the same material after taking ten cycles of ML readouts and annealing at 200 °C, (d) ML glow curves of the same material after taking TL readouts and annealing at 200 °C, (e) the ML glow curves of the material annealed at 800 ^o^C in reducing (10% H_2_ in Ar) atmosphere, (f) the ML glow curves of the material firstly annealed at 800 °C in air and later annealing at 400 °C in reducing (10% H_2_ in Ar) atmosphere, (g) the ML glow curves of the material firstly annealed at 800 °C in air and later annealing at 600 °C in reducing (10% H_2_ in Ar) atmosphere, (h) the ML glow curves of the material firstly annealed at 800 °C in air and later annealing at 800 °C in reducing (10% H_2_ in Ar) atmosphere. Applied load variation with time has also been shown here; (B) all the experiments described are also done for nanocrystalline materials also. Typical results, for different temperatures for the material having ∼32 nm particle size, are shown in this figure except for annealing at 400 and 600 °C as not much change in ML intensity was observed earlier.

**Fig. 19:**
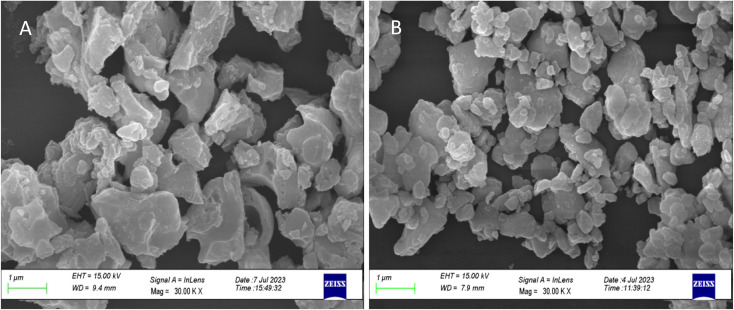
FESEM of the nanomaterial (32 nm) annealed at 800 °C: (A) in air atmosphere and (B) in reducing (10% H_2_ in Ar) atmosphere for 1.0 h.

The regeneration of traps in a phosphor material (whether self-regeneration or regeneration by some simple method) is very important for its reuse and application point of view. For example, a ML phosphor is used for its application for knowing the condition of an old bridge or a monument of historical importance, and a film or a paint is applied at some key places of the structures. If a crack is being developed, the ML could be visualized to see the propagation of the crack. But in this process, the traps/LCs already stored would be utilized, and over a period, there would be fading of the ML and would need their regeneration. Dosimetry of UV or high-energy radiation could also be done using this process. The regeneration of traps could be done by either annealing the material between 80 to 300 °C or by irradiating it with UV or other high-energy radiations.^86–88^ Such studies have also been done here to understand the phenomena in more details. In the present study, the annealing/emptying of traps was done in two ways; firstly, TL of the material was taken up to 220 °C with the linear heating rate of 5 °C s^−1^ by which all the traps get destroyed by recombinations. In the second method, ML was taken repeatedly (up to 10 cycles) to ensure no traps (negligible amount) were left inside. A part of the material was irradiated for different time intervals of UV-radiation (350 nm radiation, 10 mW at the sample) and ML was taken. The materials annealed in air (at 200 °C) and in reducing (10% H_2_ in Ar) atmosphere at different temperatures (400, 600, and 800 °C) were used to take ML. The results are shown in Fig. 17(A and B). It could be seen in the figure that the intensity of the UV-irradiated samples goes on increasing with the irradiation time, showing that traps could be regenerated using UV radiation. The intensity was around 5 times more than that of the initial (annealed at 200 °C for 1.0 h). This shows that more traps are generated due to redox reactions/charge transfer. The material, after repeated ML (10 cycles) was annealed at 200 °C in air and ML was taken. The intensity of this sample was found to be half of that during the first cycle. This shows that all the traps could not be regenerated by annealing due to two reasons; firstly, as mentioned earlier, there may be phase transition occurring while taking ML and after releasing the load, the material might not have recovered fully to its original phase. Secondly, there could be redox reactions during ML and annealing leading to loss of traps/dislocations resulting in less ML. In another experiment, the ML of the material annealed in a reducing atmosphere at 800 °C was taken repeatedly (10 cycles) to destroy all the traps. The material was annealed at different temperatures in the same (reducing atmosphere) for 1.0 h and ML was taken. It could be seen in Fig. 18, (Fig. 18a for microcrystalline material and Fig. 18b for the nanocrystalline material), that for the sample annealed at 400 °C, no ML was observed, and for 600 °C also, no ML was seen; however, for the sample annealed at 800 °C for 1.0 h, the ML was around 2 times more than that of the first cycle before annealing the second time. The same trend was found for micro- as well as nanomaterials. This clearly shows that the regeneration of traps does not depend only on the redox reactions that are taking place while annealing in a reducing atmosphere but also mainly on the phase transition occurring at around 800 °C as there might be getting more dislocations generated due to the movement of the lattice planes. It was also found that the nanoparticles not only change their morphology but also grow in size (Fig. 19) and turn out to be of micro size in both the cases of annealing the nanomaterial (∼32 nm), annealed in air and reducing atmosphere. However, in the former case, the ML intensity diminishes, while in the latter, it becomes the most sensitive. This is possible only due to redox reactions and phase change. As mentioned earlier, in the former case, most of the Eu-ions might have been oxidized to Eu^3+^ and formed some complex or some other phase reducing number of traps responsible for ML, while in the latter case, the reduction of most of the Eu^3+^ ions to Eu^2+^ would have enhanced the ML to the highest sensitivity (Fig. 18b). This is very important from the application point of view that since EML in polymer films are used for stress sensors, the question of phase transition would not arise and it is good that even if there is temperature fluctuations, sensing will not be much affected and the only options for regeneration of the traps in such sensors could be done by UV-radiation.

## 4 Conclusions

Eu-doped SrAl_2_O_4_ phosphor material was successfully synthesized by combustion method using nitrate salts of strontium, aluminum and europium (as impurity) and urea as fuel. The material of different particle size ranges was obtained in microcrystalline forms by crushing and sieving through standard test sieves and in nanocrystalline forms of different particle size ranges through ball milling for different time intervals. The EML and impulsive ML studies as well as TL and OSL studies show that there is direct correlation between the particle size and the luminescence intensity from the material and goes on decreasing with the particle size. This is because the compressive EML or impulsive ML not only depend on the number of traps but also the number of dislocations, their lengths, and movements. The length and movement of the dislocations may not generate a sufficient local voltage in the case of nanoparticles to excite the impurity ions decreasing the luminescence intensity. The kind of traps having different activation energies (trap depths) have been identified by the TL studies. It was also observed that the annealing atmosphere, its temperature, redox reactions, and phase transitions play important roles in the generation and regeneration of traps. Traps could be generated by heating the material in a reducing atmosphere at around 800 °C (phase transition temperature). However, it is also easier to generate and regenerate traps by irradiating to UV-radiation than by heating. The particle size effect has great importance in fabricating stress sensors.

## Author contributions

The original idea was conceived by the corresponding author (PDS), funds were managed, facilities were developed, paper was written and edited by PDS. Experimental work was done by the co-author (Lucky) under the supervision of PDS.

## Conflicts of interest

There is no conflict of interest to declare.

## Supplementary Material

RA-013-D3RA02514D-s001
