# Supplementary material for: Mechanoluminescence, thermoluminescence, optically stimulated luminescence and photoluminescence in SrAl2O4:Eu micro- and nanophosphors: effect of particle size and annealing in different atmospheres
Source: RSC Adv. 2023 Aug 29;13(36):25579–98. doi: 10.1039/d3ra02514d (PMC10463121; doi:10.1039/d3ra02514d)

Electronic Supplementary Material (ESI) for RSC Advances.
This journal is © The Royal Society of Chemistry 2023

Mechanoluminescence (ML), Thermoluminescence (TL), Optically stimulated luminescence (OSL) and photoluminescence (PL) in $SrAl_2O_4$:Eu nanophosphor: effects of particle size and annealing in different atmospheres

Lucky Sharma and P D Sahare*

Department of Physics & Astrophysics, University of Delhi, Delhi 110 007

*Corresponding author Email: pdsahare@physics.du.ac.in

Supplementary data

Figure S-1: Experimental set up for taking ML by applying pressure with constant pressing rate. All different parts of the equipment are listed in the inset.

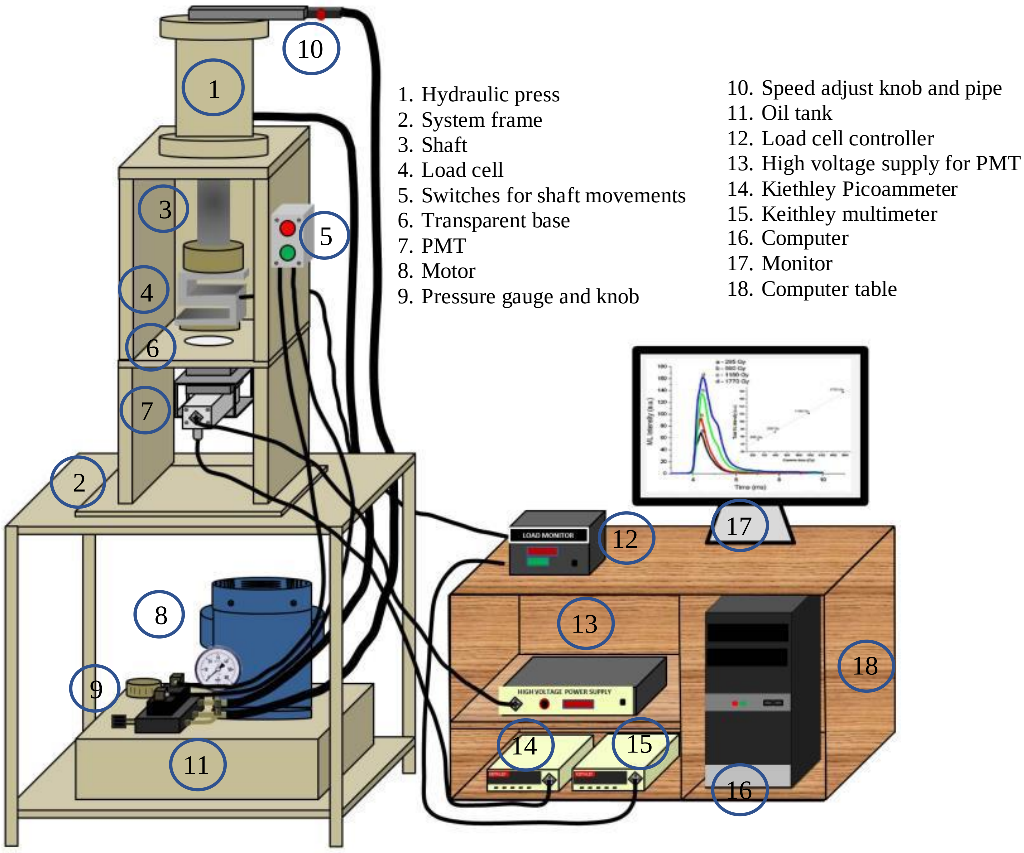

Figure S-2: Rietveld refinement of a typical XRD pattern of the SAOE ML phosphor. Experimental data has also been shown for comparison. Very good fit between the simulated pattern and the experimental one could be seen in the figure.

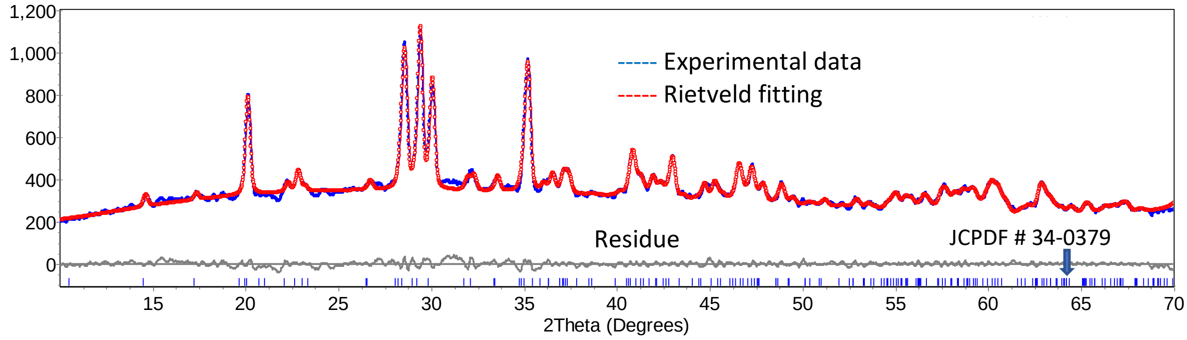

Figs. S-3(A), S-3(B-F), respectively, for particles in the particle size range of 250-150 μm. The EDS spectrum is shown in Fig S-3(G) and a table consisting of the composition of the material in atomic% and weight% is also shown in its inset. Similar data for all the other particle sizes is also shown in Figs. S-4(A), S-4(B-F) and S-4(G), Figs. S-5(A), S-5(B-F) and S-5(G), Figs. S-6(A), S-6(B-F) and S-6(G), Figs. S-7(A), S-7(B-F) and S-7(G), Figs. S-8(A), S-8(B-F) and S-8(G), Figs. S-9(A), S-9(B-F) and S-9(G) and Figs. Figs. S-10(A), S-10(B-F) and S-10(G) for the average particle sizes 150-105, 105-75, 75-45, 0.55 μm, 77 nm, 47 nm and 32 nm, respectively.

Fig. S-3

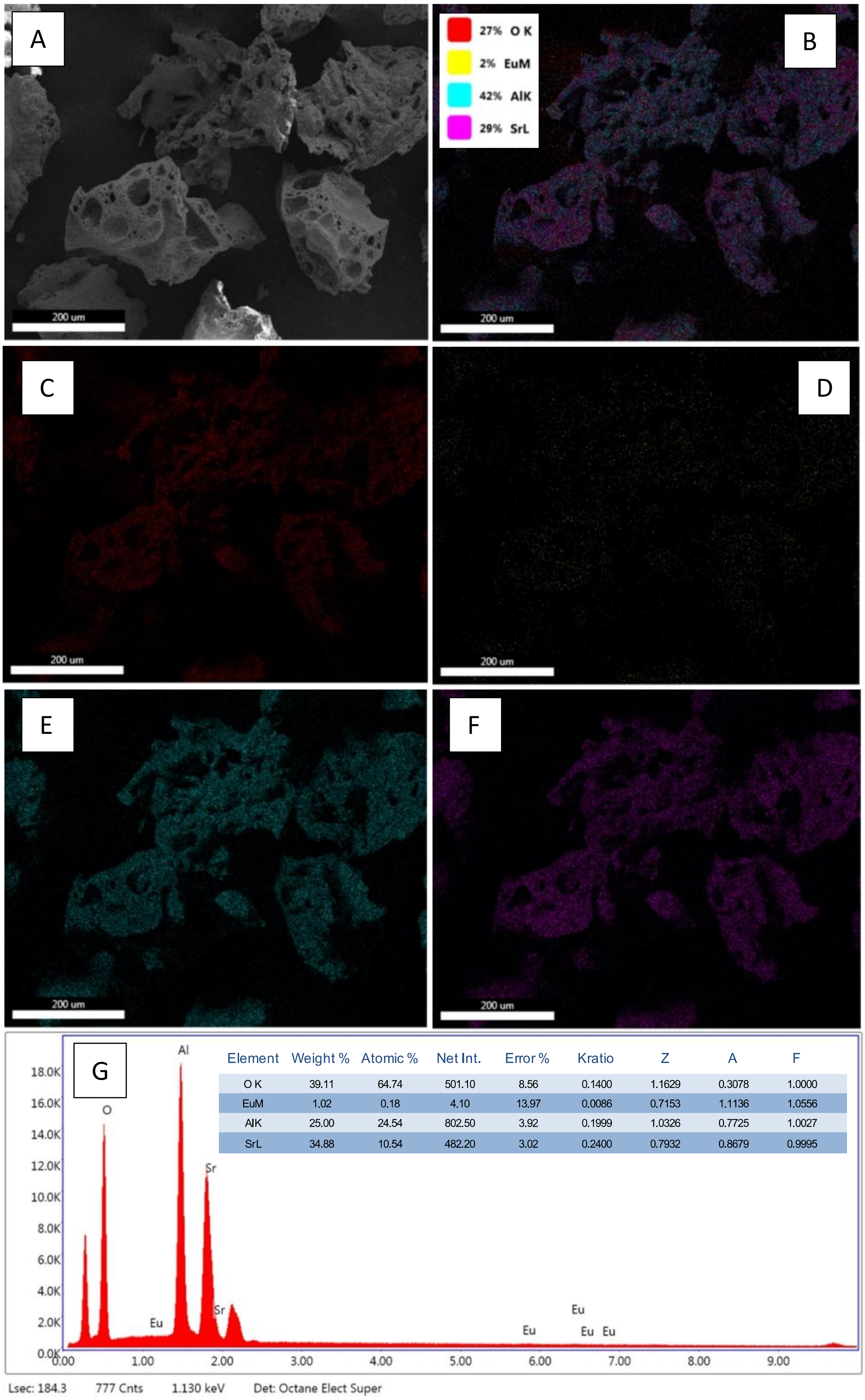
A
B
27% O K
2% EuM
42% AlK
29% SrL
C
D
E
F
200 um
G
Element Weight % Atomic % Net Int. Error % Kratio Z A F
O K 39.11 64.74 501.10 8.56 0.1400 1.1629 0.3078 1.0000
EuM 1.02 0.18 4.10 13.97 0.0086 0.7153 1.1136 1.0556
AlK 25.00 24.54 802.50 3.92 0.1999 1.0326 0.7725 1.0027
SrL 34.88 10.54 482.20 3.02 0.2400 0.7932 0.8679 0.9995
O
Al
Sr
Eu
0.0K 2.0K 4.0K 6.0K 8.0K 10.0K 12.0K 14.0K 16.0K 18.0K
0.00 1.00 2.00 3.00 4.00 5.00 6.00 7.00 8.00 9.00
Lsec: 184.3 777 Cnts 1.130 keV Det: Octane Elect Super

Fig. S-4

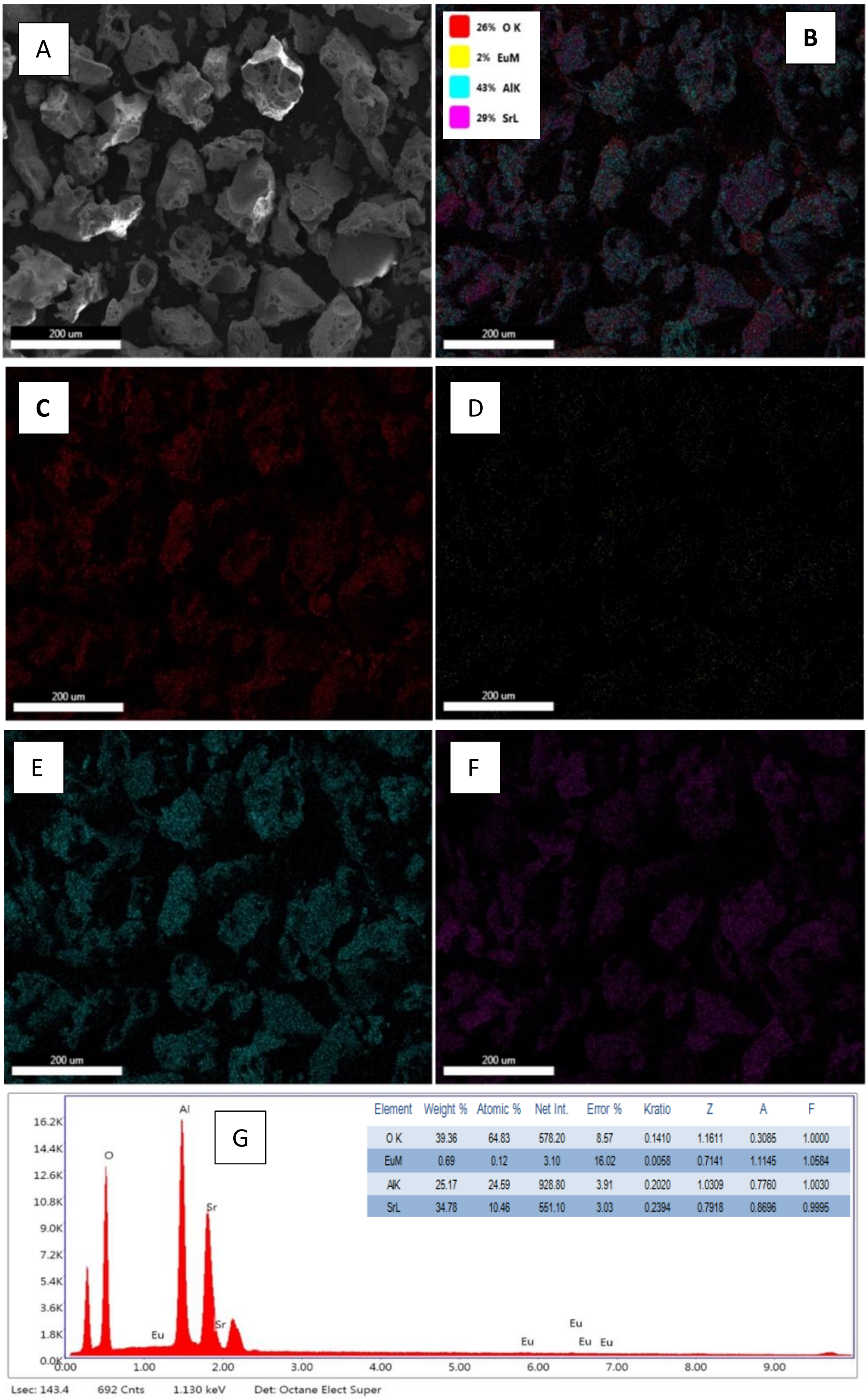

Element	Weight %	Atomic %	Net Int.	Error %	Kratio	Z	A	F
O K	39.36	64.83	578.20	8.57	0.1410	1.1611	0.3085	1.0000
EuM	0.69	0.12	3.10	16.02	0.0058	0.7141	1.1145	1.0584
AlK	25.17	24.59	928.80	3.91	0.2020	1.0309	0.7760	1.0030
SrL	34.78	10.46	551.10	3.03	0.2394	0.7918	0.8696	0.9995

Fig. S-5

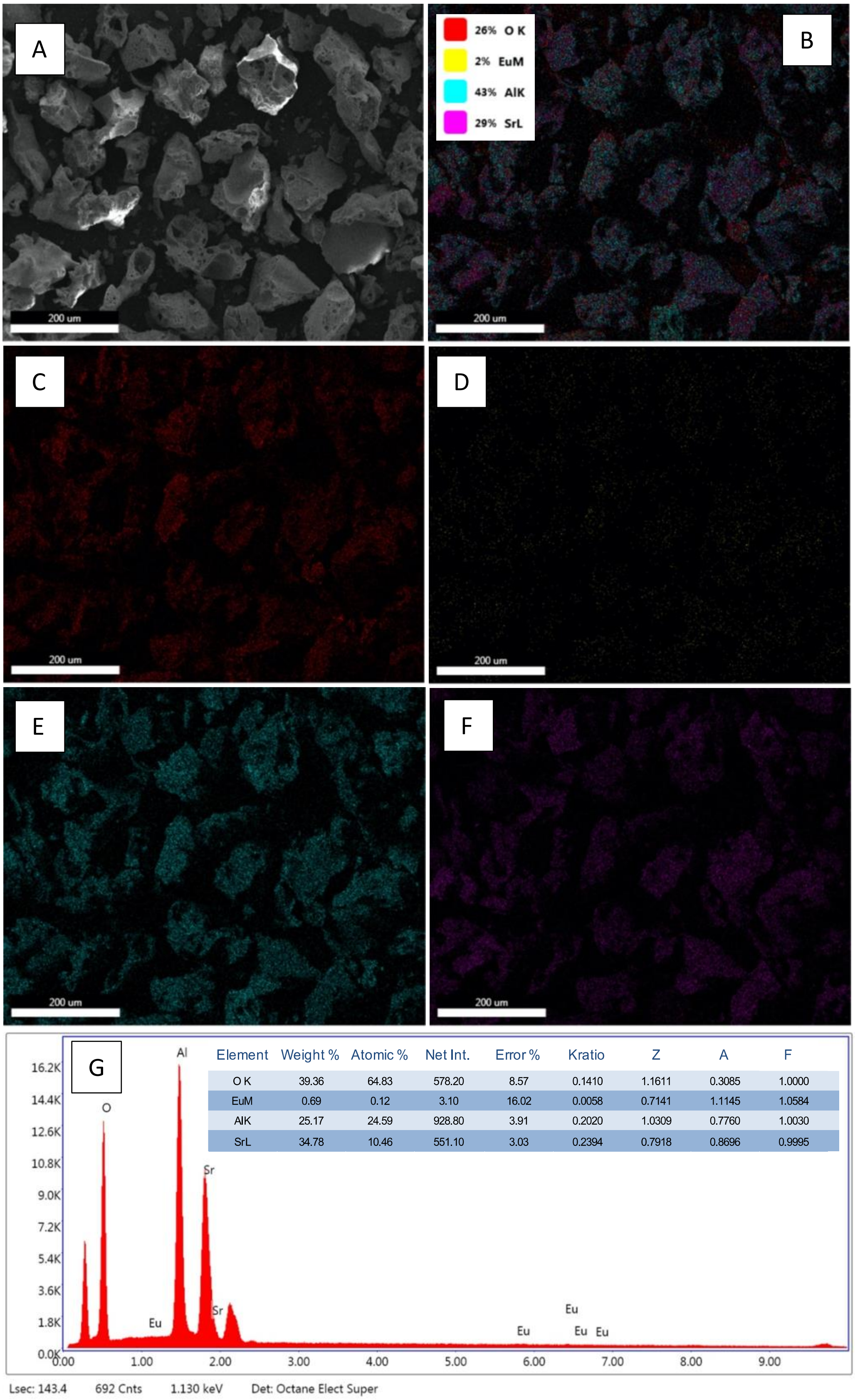
A
B
26% O K
2% EuM
43% AlK
29% SrL
C
D
E
F
G
200 um
Element | Weight % | Atomic % | Net Int. | Error % | Kratio | Z | A | F
O K | 39.36 | 64.83 | 578.20 | 8.57 | 0.1410 | 1.1611 | 0.3085 | 1.0000
EuM | 0.69 | 0.12 | 3.10 | 16.02 | 0.0058 | 0.7141 | 1.1145 | 1.0584
AlK | 25.17 | 24.59 | 928.80 | 3.91 | 0.2020 | 1.0309 | 0.7760 | 1.0030
SrL | 34.78 | 10.46 | 551.10 | 3.03 | 0.2394 | 0.7918 | 0.8696 | 0.9995
Al
O
Sr
Eu
16.2K
14.4K
12.6K
10.8K
9.0K
7.2K
5.4K
3.6K
1.8K
0.0K
0.00 1.00 2.00 3.00 4.00 5.00 6.00 7.00 8.00 9.00
Lsec: 143.4 692 Cnts 1.130 keV Det: Octane Elect Super

Particle size: S-6

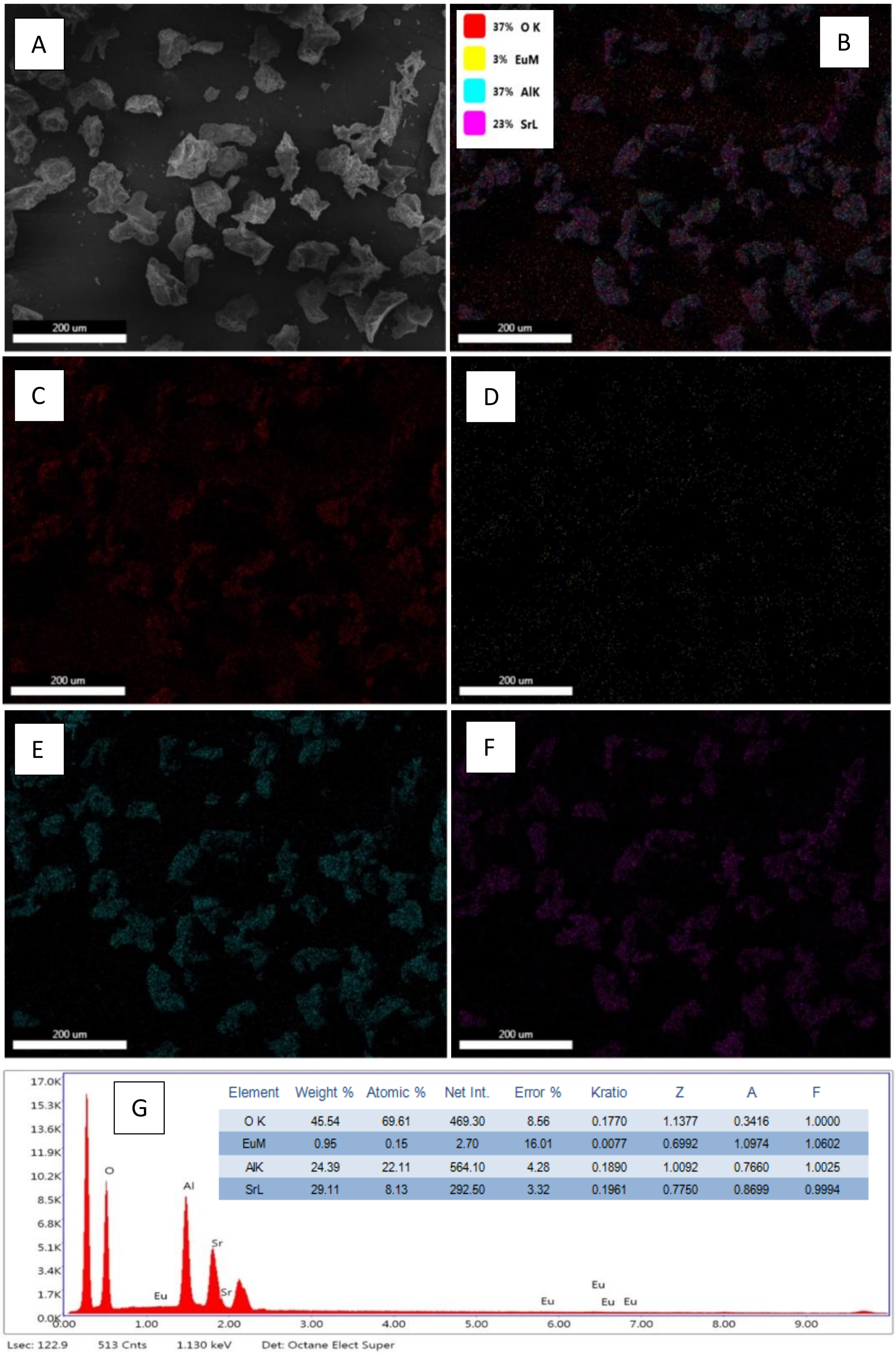

Element	Weight %	Atomic %	Net Int.	Error %	Kratio	Z	A	F
O K	45.54	69.61	469.30	8.56	0.1770	1.1377	0.3416	1.0000
EuM	0.95	0.15	2.70	16.01	0.0077	0.6992	1.0974	1.0602
AlK	24.39	22.11	564.10	4.28	0.1890	1.0092	0.7660	1.0025
SrL	29.11	8.13	292.50	3.32	0.1961	0.7750	0.8699	0.9994

Fig. S-7

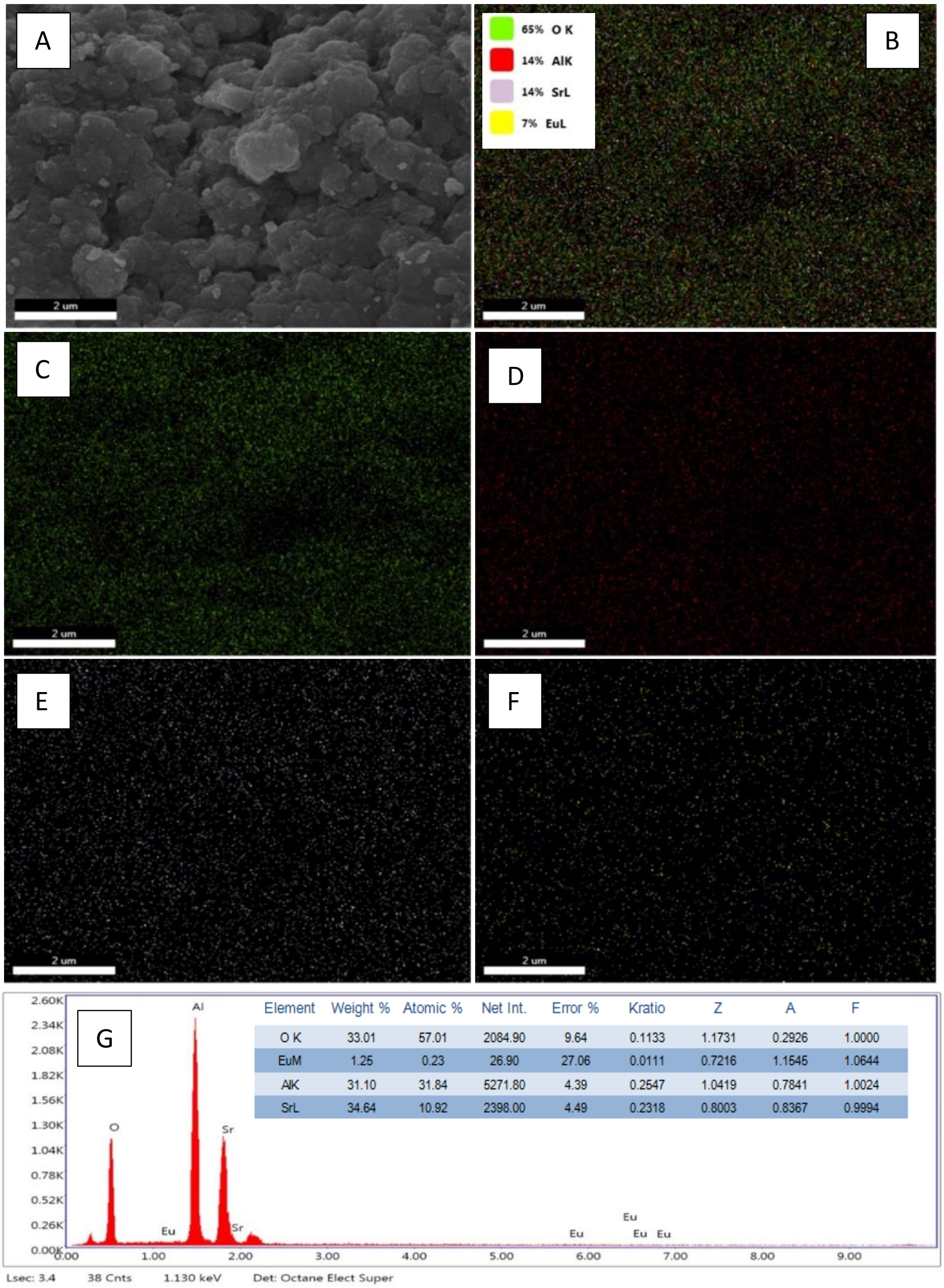
A
B
65% O K
14% AlK
14% SrL
7% EuL
C
D
E
F
2 um
G
Element Weight % Atomic % Net Int. Error % Kratio Z A F
O K 33.01 57.01 2084.90 9.64 0.1133 1.1731 0.2926 1.0000
EuM 1.25 0.23 26.90 27.06 0.0111 0.7216 1.1545 1.0644
AlK 31.10 31.84 5271.80 4.39 0.2547 1.0419 0.7841 1.0024
SrL 34.64 10.92 2398.00 4.49 0.2318 0.8003 0.8367 0.9994
O
Al
Sr
Eu
Lsec: 3.4 38 Cnts 1.130 keV Det: Octane Elect Super

Fig. S-8

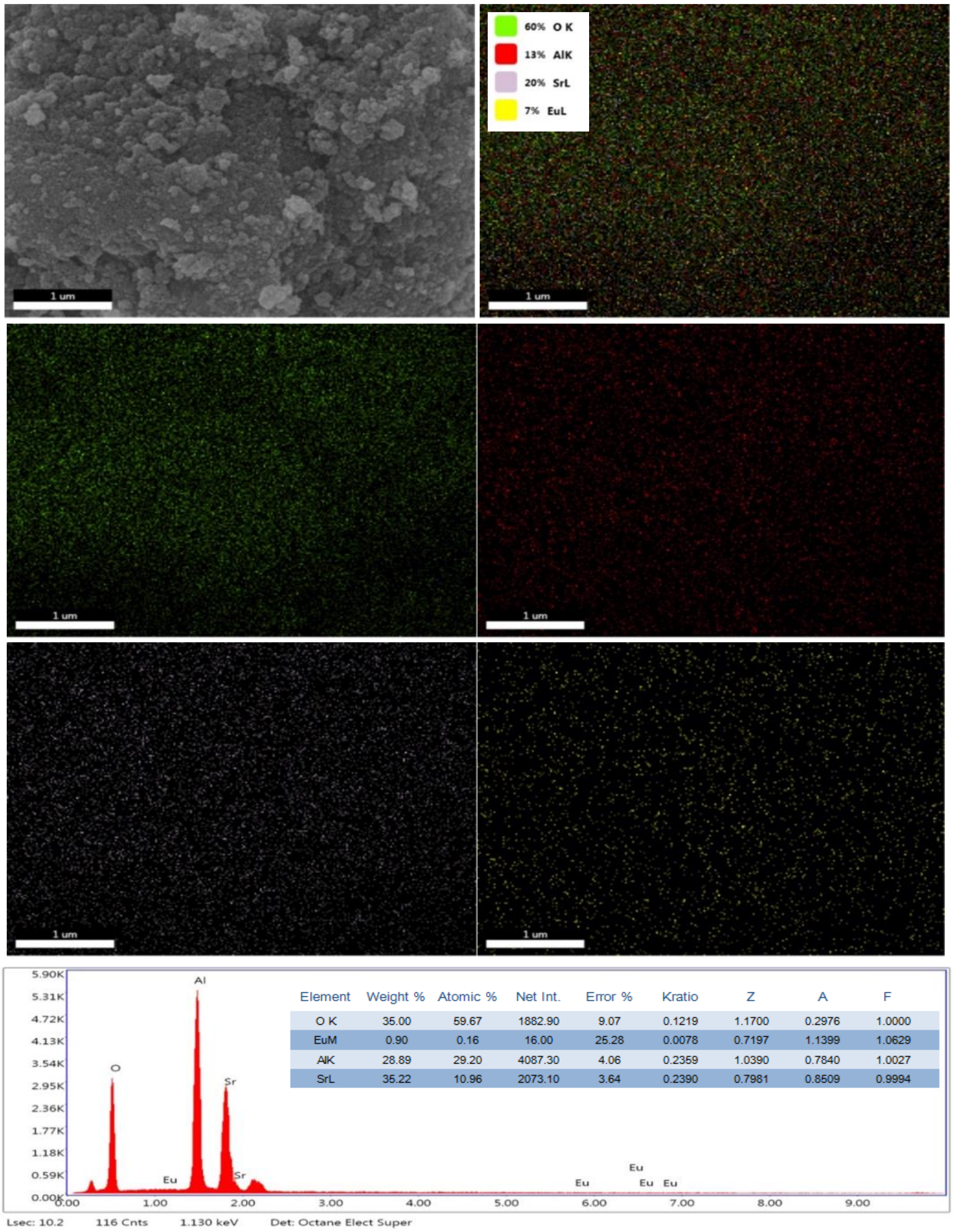

Element	Weight %	Atomic %	Net Int.	Error %	Kratio	Z	A	F
O K	35.00	59.67	1882.90	9.07	0.1219	1.1700	0.2976	1.0000
EuM	0.90	0.16	16.00	25.28	0.0078	0.7197	1.1399	1.0629
AlK	28.89	29.20	4087.30	4.06	0.2359	1.0390	0.7840	1.0027
SrL	35.22	10.96	2073.10	3.64	0.2390	0.7981	0.8509	0.9994

Fig. S-9

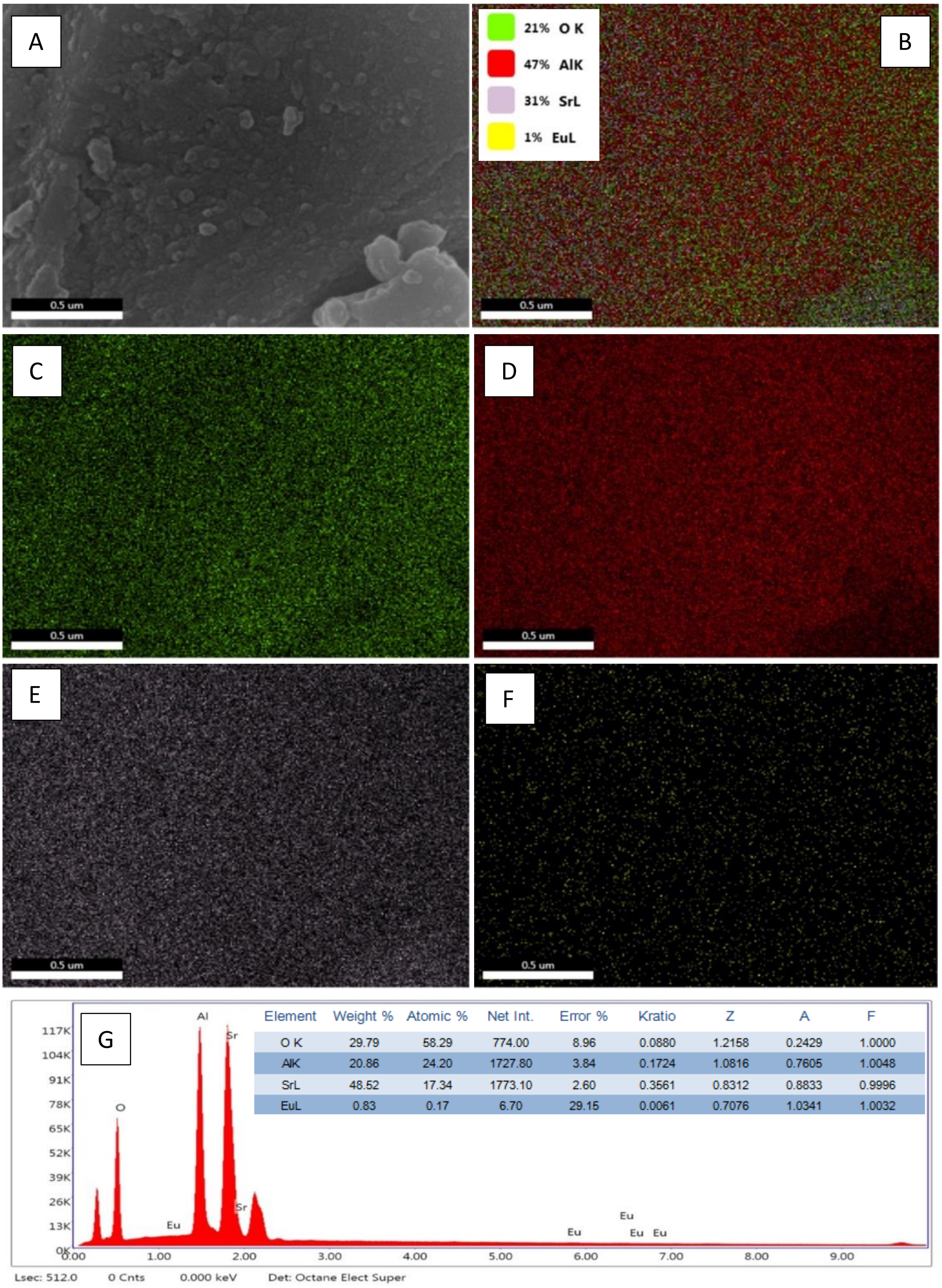

Element	Weight %	Atomic %	Net Int.	Error %	Kratio	Z	A	F
O K	29.79	58.29	774.00	8.96	0.0880	1.2158	0.2429	1.0000
AlK	20.86	24.20	1727.80	3.84	0.1724	1.0816	0.7605	1.0048
SrL	48.52	17.34	1773.10	2.60	0.3561	0.8312	0.8833	0.9996
EuL	0.83	0.17	6.70	29.15	0.0061	0.7076	1.0341	1.0032

Fig. S-10

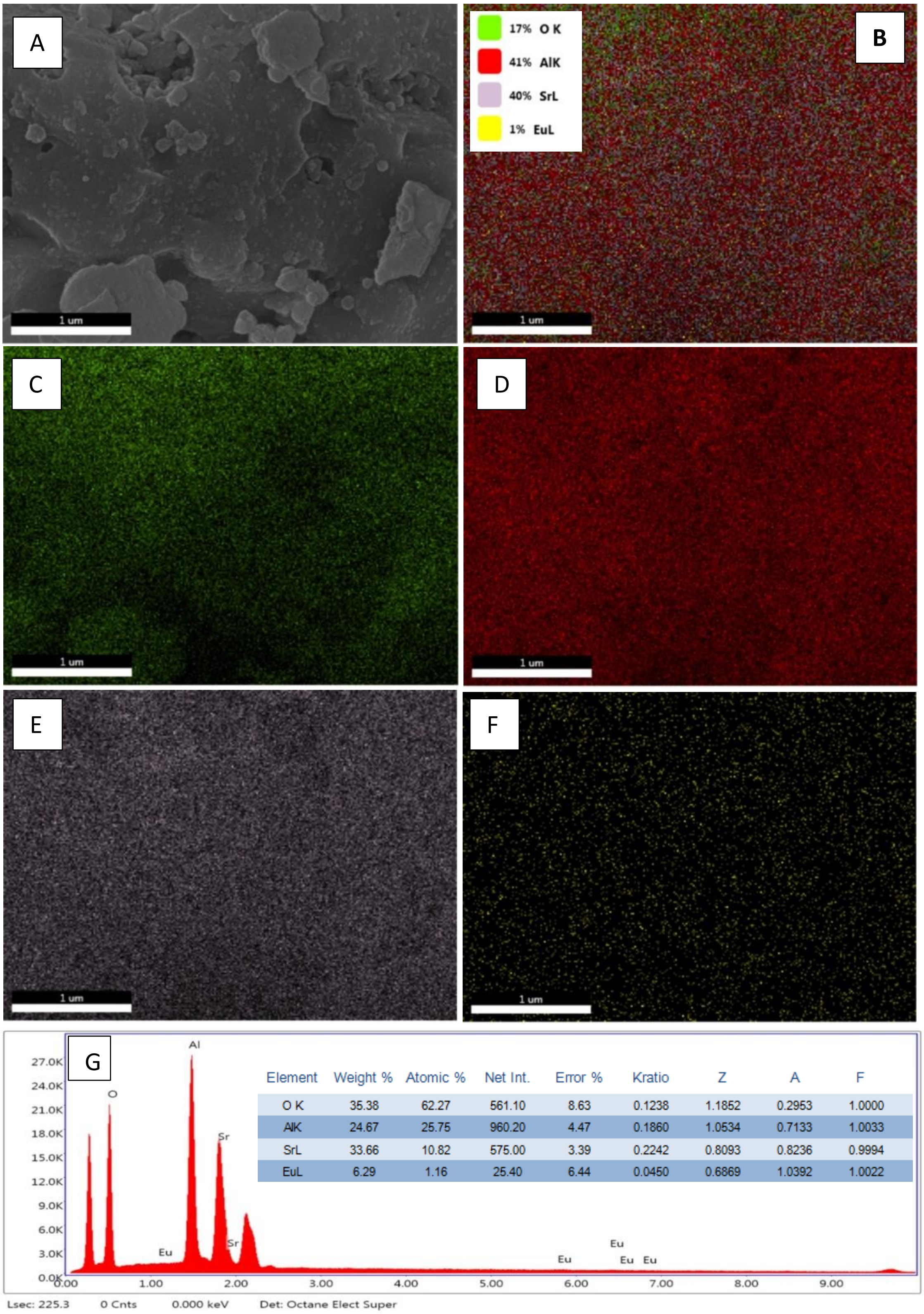

Element	Weight %	Atomic %	Net Int.	Error %	Kratio	Z	A	F
O K	35.38	62.27	561.10	8.63	0.1238	1.1852	0.2953	1.0000
AlK	24.67	25.75	960.20	4.47	0.1860	1.0534	0.7133	1.0033
SrL	33.66	10.82	575.00	3.39	0.2242	0.8093	0.8236	0.9994
EuL	6.29	1.16	25.40	6.44	0.0450	0.6869	1.0392	1.0022

Fig. S-11: Effect of impurity concentration on the ML peak intensity of $SrAl_2O_4$: Eu phosphor material. The variation of the ML peak intensity with impurity concentration is also shown in the inset. The maximum ML peak intensity was found to be for 1.5 mol% impurity concentration.

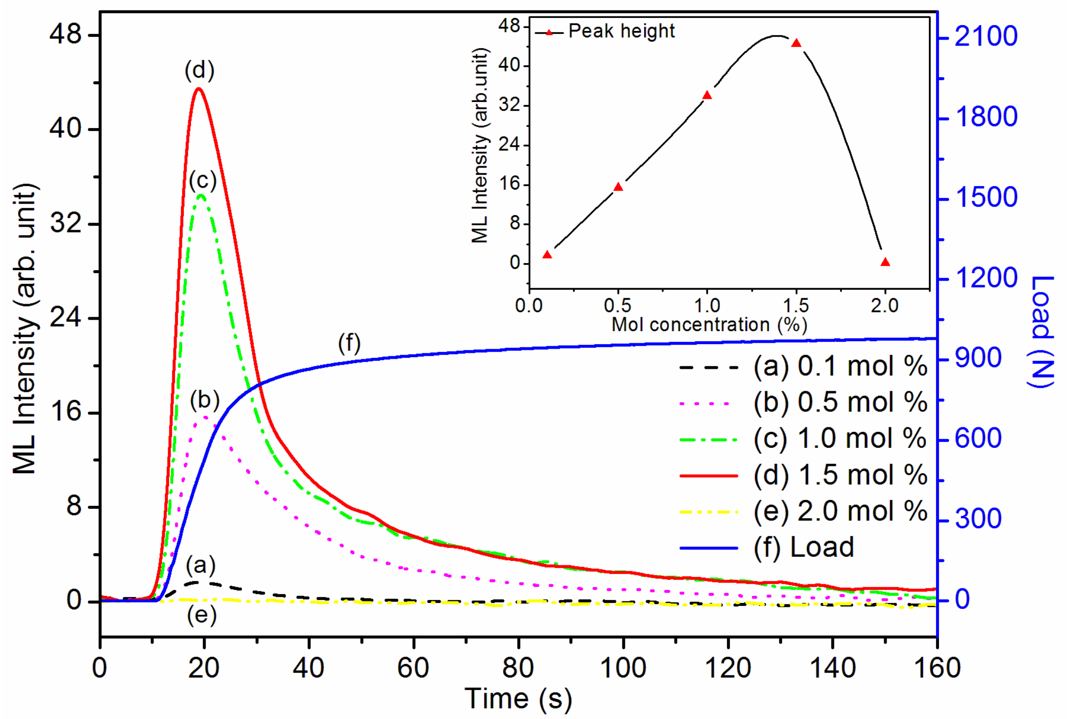

Fig. S-12: ML glow curves of SAOE phosphor after annealing in air. The variation of the EML intensity with the annealing temperature is also shown in the inset.

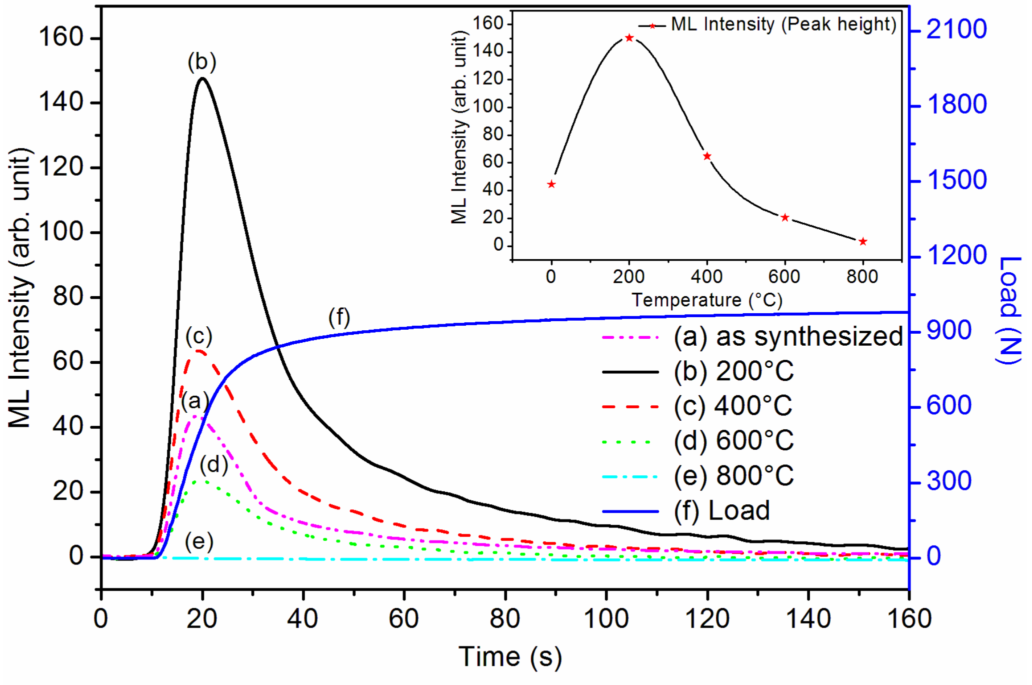

Fig. S-13: Determination of induced strain in nanomaterials of different particles due to ball milling: a) 550 nm, b) 77 nm, c) 47 nm, d) 32 nm.

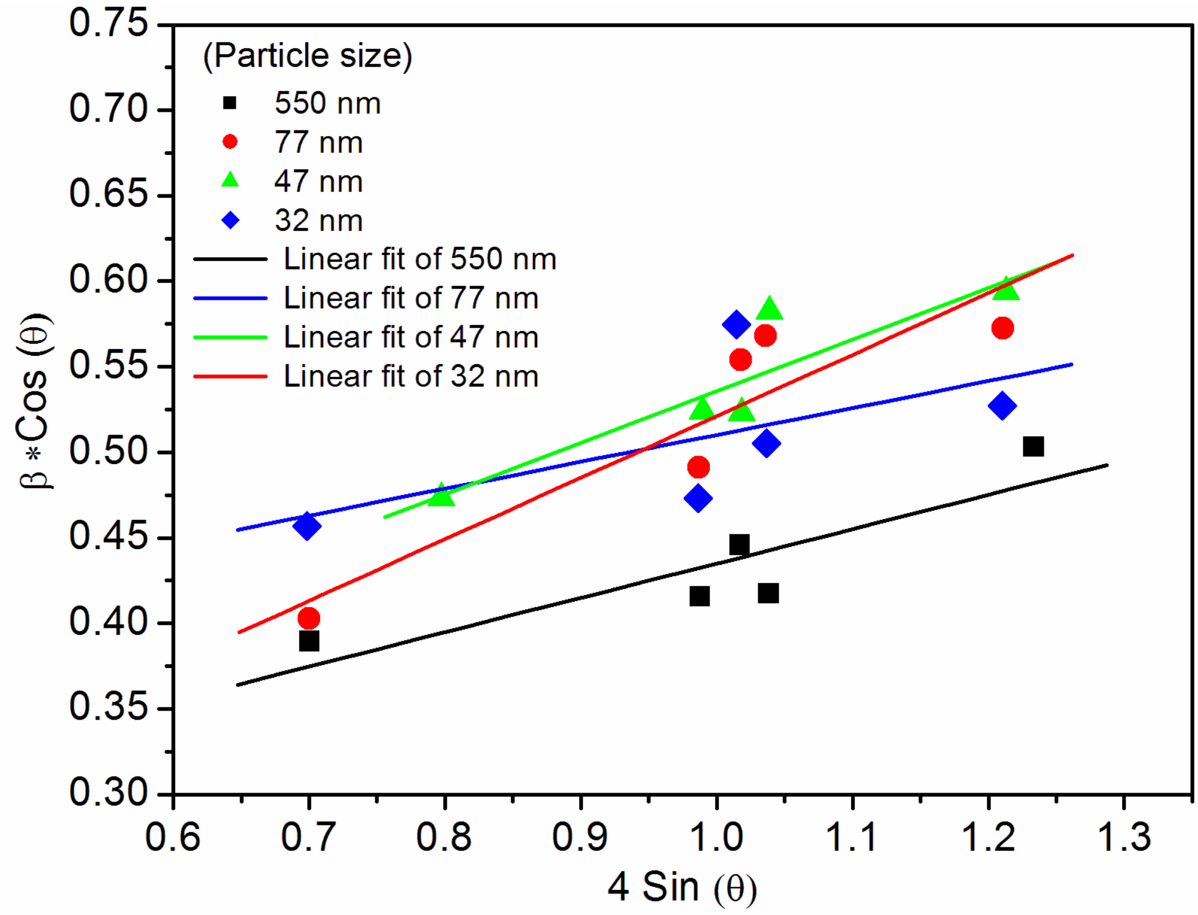

Supplement: RA-013-D3RA02514D-s001 [file RA-013-D3RA02514D-s001.pdf]
